# A DNA methylation‐based definition of biologically distinct breast cancer subtypes

**DOI:** 10.1016/j.molonc.2014.10.012

**Published:** 2014-11-05

**Authors:** Olafur A. Stefansson, Sebastian Moran, Antonio Gomez, Sergi Sayols, Carlos Arribas-Jorba, Juan Sandoval, Holmfridur Hilmarsdottir, Elinborg Olafsdottir, Laufey Tryggvadottir, Jon G. Jonasson, Jorunn Eyfjord, Manel Esteller

**Affiliations:** ^1^ Cancer Epigenetics and Biology Program (PEBC), Bellvitge Biomedical Research Institute, L'Hospitalet, Barcelona, Catalonia 08908, Spain; ^2^ The Cancer Research Laboratory, Medical Faculty, University of Iceland, Reykjavik, Iceland; ^3^ The Icelandic Cancer Registry, Reykjavik, Iceland; ^4^ Department of Pathology, Landspitali University Hospital, Reykjavik, Iceland; ^5^ Department of Physiological Sciences II, School of Medicine, University of Barcelona, Barcelona, Catalonia, Spain; ^6^ Institucio Catalana de Recerca i Estudis Avançats (ICREA), Barcelona, Catalonia, Spain

**Keywords:** Breast cancer, DNA methylation, Microarrays, Biological subtypes, Prognosis

## Abstract

In cancer, epigenetic states are deregulated and thought to be of significance in cancer development and progression. We explored DNA methylation‐based signatures in association with breast cancer subtypes to assess their impact on clinical presentation and patient prognosis. DNA methylation was analyzed using Infinium 450K arrays in 40 tumors and 17 normal breast samples, together with DNA copy number changes and subtype‐specific markers by tissue microarrays. The identified methylation signatures were validated against a cohort of 212 tumors annotated for breast cancer subtypes by the PAM50 method (The Cancer Genome Atlas). Selected markers were pyrosequenced in an independent validation cohort of 310 tumors and analyzed with respect to survival, clinical stage and grade. The results demonstrate that DNA methylation patterns linked to the luminal‐B subtype are characterized by CpG island promoter methylation events. In contrast, a large fraction of basal‐like tumors are characterized by hypomethylation events occurring within the gene body. Based on these hallmark signatures, we defined two DNA methylation‐based subtypes, Epi‐LumB and Epi‐Basal, and show that they are associated with unfavorable clinical parameters and reduced survival. Our data show that distinct mechanisms leading to changes in CpG methylation states are operative in different breast cancer subtypes. Importantly, we show that a few selected proxy markers can be used to detect the distinct DNA methylation‐based subtypes thereby providing valuable information on disease prognosis.

## Highlights

- We describe distinct signatures associated with luminal‐B and basal‐like subtypes.
- The signatures identified show differences in genes, but also in the CpG context.
- A selected set of proxy markers for each signature revealed their clinical relevance.

## 1 Introduction

Breast cancer is the most common cancer among women and ranks among the leading causes of cancer‐related deaths (Hortobagyi et al., 2005). Nevertheless, the over all prognosis of breast cancer patients has been improving over time, with marked changes seen since the introduction of adjuvant chemotherapy and ionizing radiation, coupled with the use of tamoxifen for patients with hormone receptor‐positive tumors and, more recently, trastuzumab for those displaying overexpression and amplification of the HER‐2 oncogene (López‐Tarruella and Martín, 2009). Further improvements in treatment of breast cancer patients are likely to emerge from our greater understanding of why only some patients develop aggressive disease and require adjuvant chemotherapy. A major milestone on the way to this goal is the definition of five biologically and clinically meaningful breast cancer subtypes based on genome‐wide expression analyses: Luminal‐A, Luminal‐B, HER‐2, Normal‐like and Basal‐like (Sorlie et al., 2003; Perou and Børresen‐Dale, 2011). An integrated analysis of genome‐wide gene expression and copy number changes was recently carried out to further refine the previously established breast cancer subtypes (Curtis et al., 2012).

Different breast cancer subtypes are thought to arise through different “evolutionary paths” reflecting distinct patterns of mutated cancer genes (Stephens et al., 2012; Cancer Genome Atlas Network, 2012; Saal et al., 2008; Chin et al., 2006; Diaz‐Cruz et al., 2010; Curtis et al., 2012). Less is known about the contribution of epigenetic changes to the development of biologically distinct breast cancer subtypes. The epigenome‐wide studies so far published on this subject have either lacked comprehensive profiling technologies or have not validated any clinical relevance in independent patient cohorts (Cancer Genome Atlas Network, 2012; Holm et al., 2010; Bediaga et al., 2010; Fang et al., 2011; Dedeurwaerder et al., 2011; Hon et al., 2012). Recent efforts to sequence cancer genomes, including those of breast cancer, have led to the identification of novel cancer genes and previously unrecognized signatures of mutational processes (Stephens et al., 2012; Cancer Genome Atlas Network, 2012). A recurring theme emerging from these studies is that acquired mutations often affect genes involved in regulating chromatin dynamics or the processing of epigenetic marks as seen in various cancer types (Stephens et al., 2012; Cancer Genome Atlas Network, 2012; Jones et al., 2010; Mamo et al., 2012). This highlights the importance the epigenome in cancer development and opens up new potentials for identifying patterns of potential relevance to patient prognosis and personalized medicine (Stefansson and Esteller, 2013; Heyn and Esteller, 2012).

## 2 Materials and methods

### 2.1 DNA samples

The study material consisted of 350 primary invasive breast tumors and 25 normal breast tissue samples obtained adjacent to tumor lesions of patients. The samples were obtained from the Department of Pathology, University Hospital, Iceland. The normal breast tissue samples obtained from the subset of 25 patients were derived from regions adjacent to the site of tumor growth. DNA was isolated by the commonly used phenol‐chloroform/proteinase‐K method. Patient information about the breast cancer samples came from the population‐based Icelandic Cancer Registry (Sigurdardottir et al., 2012). Data on clinical staging and histological grade from the Icelandic Cancer Registry were based on analyses by pathologists at the Department of Pathology, Landspitali University Hospital. For clinical staging, the TNM system was followed (tumor size and nodal status), with histological grade assessed according to the Nottingham system. Informed consent was obtained from all patients and all work was carried out according to permissions from the Icelandic Data Protection Commission (2006050307) and Bioethics Committee (VSNb2006050001/03‐16).

### 2.2 DNA methylation analysis

Infinium HumanMethylation450 BeadChips were used to analyze DNA methylation on a genome‐wide scale in the discovery cohort (40 tumors and 17 normal breast tissues). The 450K Infinium microarray data tissue samples have been deposited to NCBI's GEO database and can be downloaded from the following link:

http://www.ncbi.nlm.nih.gov/geo/query/acc.cgi?token=enijueysntsbvyd&acc=GSE52865.

The experimental procedures used were those recommended by the manufacturer (Supplementary Materials & methods).

The PyroMark Q96 system for pyrosequencing was used to assess selected markers in the validation cohort. The primer sequences used in this analysis were designed using Qiagen's Pyromark Assay Design 2·0 software (details and primer sequences are available in the Supplementary Methods).

### 2.3 Statistical analyses & bioinformatics procedures

Cluster analysis (carried out in Section 3.1) of the epigenome‐wide data was performed using unsupervised hierarchical clustering with complete linkage and Manhattan distance on the top 5000 CpG's found differentially methylated between breast tumors and normal breast samples (see further in Supplementary Methods). We then used the pvclust package in R to define statistically significant tumor clusters indicating patterns of potential biological relevance. The DNA methylation data were then analyzed in a supervised design to identify sites associated with expression‐based subtypes using the SAMr uni‐variate multi‐class marker discovery procedure (samr package in R) wherein permutations are used to estimate the false discovery rate (FDR) (with FDR <5% considered statistically significant) as carried out in Section 3.2. In this analysis, we additionally applied a threshold for the significant sites using mean differences on a subtype‐by‐subtype basis (also including the group of normal breast tissue samples) with minimum change of ±0.10 in beta‐values required in all subtype–subtype comparisons for each site (wherein the direction of change was required to be consistent). The CpG sequence context of differentially methylated genes between subtypes was then analyzed (carried out in Section 3.2) by hypothesis testing for differences in the counting of CpGs by categories reflecting functionally relevant sequences, i.e. 1) those located proximal to the transcription start site (TSS) (inferred as promoter regions, i.e. CpG's located within 200 b of the TSS or within the 5′UTR/1stExon), 2) those located more distantly from the TSS lying within the interval from 200 to 1500 bp upstream of the TSS site and 3) those found within the gene body (excluding the 1st Exon). More detailed analysis in this regards included the classification of CpG's within promoter regions into three distinct groups, i.e. those found within 1) CpG islands, 2) CpG shores and 3) CpG poor regions. In Section 3.3, we define DNA methylation‐based subtypes by a cross‐validation procedure that enables classification of each tumor according to the identified hallmark signatures (as defined in Section 3.2). This was carried out using a well‐known pattern recognition algorithm called prediction analysis for microarrays (PAM) in a leave‐one‐out cross‐validation design (Tibshirani et al., 2002).

Tabular data were analyzed by the two‐sided chi‐squared contingency test for count data (carried out in Section 3.6). The Kaplan–Meier method was used to generate survival curves while relative hazards were estimated in multivariate analyses using the Cox proportional hazards model (carried out in Sections 3.6, 3.7). Patients were followed from the date of diagnosis until death or last date of follow‐up (December 31st 2009). The outcome was breast cancer‐specific survival, defined as the time from diagnosis to patient death from breast cancer as registered on death certificates. Patients who died of causes other than breast cancer were censored at the date of death. Survival analyses were carried out in R (survival package). Hazard ratios and the corresponding *P*‐values are reported for each methylation‐based subtype, coded as a categorical variable, after having adjusted for the potential confounding factors of patient age and year of diagnosis.

## 3 Results

### 3.1 Genome‐wide DNA methylation patterns and breast cancer subtypes

**Figure 1:**
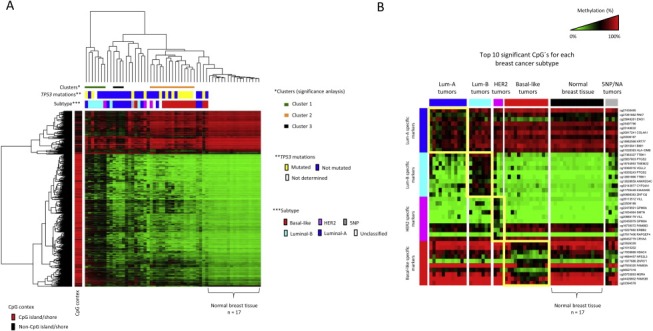
DNA methylation changes in breast tumors are non‐random and define patterns correlated with clinically and biologically relevant subtypes. A) Cluster analysis of differentially methylated CpGs between breast cancers and normal breast tissue (the top 5000 most significant CpGs). Tumor characteristics (by columns on top of the heat‐map) in terms of breast cancer subtype along with the presence of acquired mutations in the TP53 gene are displayed together with the CpG context (by rows on the left‐hand side) according to the color scheme shown at the right‐hand side and bottom of the figure, respectively. The statistically significant tumor patterns/clusters (identified by the pvclust method in R) are shown as colored bars immediately below the dendrogram. B) The top 10 significant CpG's specifically characterizing each of the four “core” subtypes are shown, i.e. the LumA, LumB, HER2 and Basal‐like subtypes. Note, 5NP (i.e. unclassified tumors due to negativity for all five phenotypic markers, i.e. ER, PR, HER2, CK5/6 and EGFR) and breast tumors with unknown subtype information are grouped together as 5NP/NA and were not included in this analysis. The normal breast tissue samples are shown and indicated in black on top of the heat‐map. Note, the heat‐map colors reflect beta‐values representing the degree of methylation from low to high as green to red, respectively (wherein black represents heterogenous/hemi‐methylation), as shown on the scale at the top‐right hand side of the figure.

CpGs that were differentially methylated between breast tumors and normal breast tissues were identified and explored in relation to biologically relevant subtypes by cluster analysis (Figure 1A; Supplementary Figure 1). This revealed a distinctive DNA methylation pattern (significant at AU > 95%; by the pvclust method) enriched with tumors of the Luminal‐B (LumB) phenotype (indicated as “Cluster 1”, Figure 1A). These tumors show extensive DNA methylation of CpG islands implying that they have acquired a methylator phenotype. Interestingly, these characteristics did not appear to be restricted to LumB tumors as a few members of other subtypes also displayed this pattern, whereby one tumor was classified as Luminal‐A (LumA) and the other as HER2 (see in “Cluster 1”; Figure 1A). The DNA methylation patterns of most, but not all, Basal‐like tumors were also distinctive (AU > 95%; indicated as “Cluster 2”, Figure 1A) in that the changes affected a different set of CpGs from those affected in most other tumors. In contrast, HER2 and Luminal‐A (LumA) breast tumors were more heterogenous in terms of their DNA methylation patterns as these tumors were more widely dispersed throughout the cluster dendrogram. Indeed, only a small subset of the LumA breast tumors (four out of twelve) showed evidence of a distinctive (i.e., statistically significant; AU > 95%) pattern of DNA methylation changes (indicated as “Cluster 3”, Figure 1A), emphasizing the biological heterogeneity within this subtype (Supplementary Figure 1). In summary, this analysis supports the notion that CpG methylation changes observed on a genome‐wide scale are systematically linked to distinct breast cancer subtypes, in particular the LumB and Basal‐like subtypes (Supplementary Table 1).

### 3.2 Distinct epigenomic characteristics between breast cancers of the Luminal‐B and basal‐like subtypes

**Figure 2:**
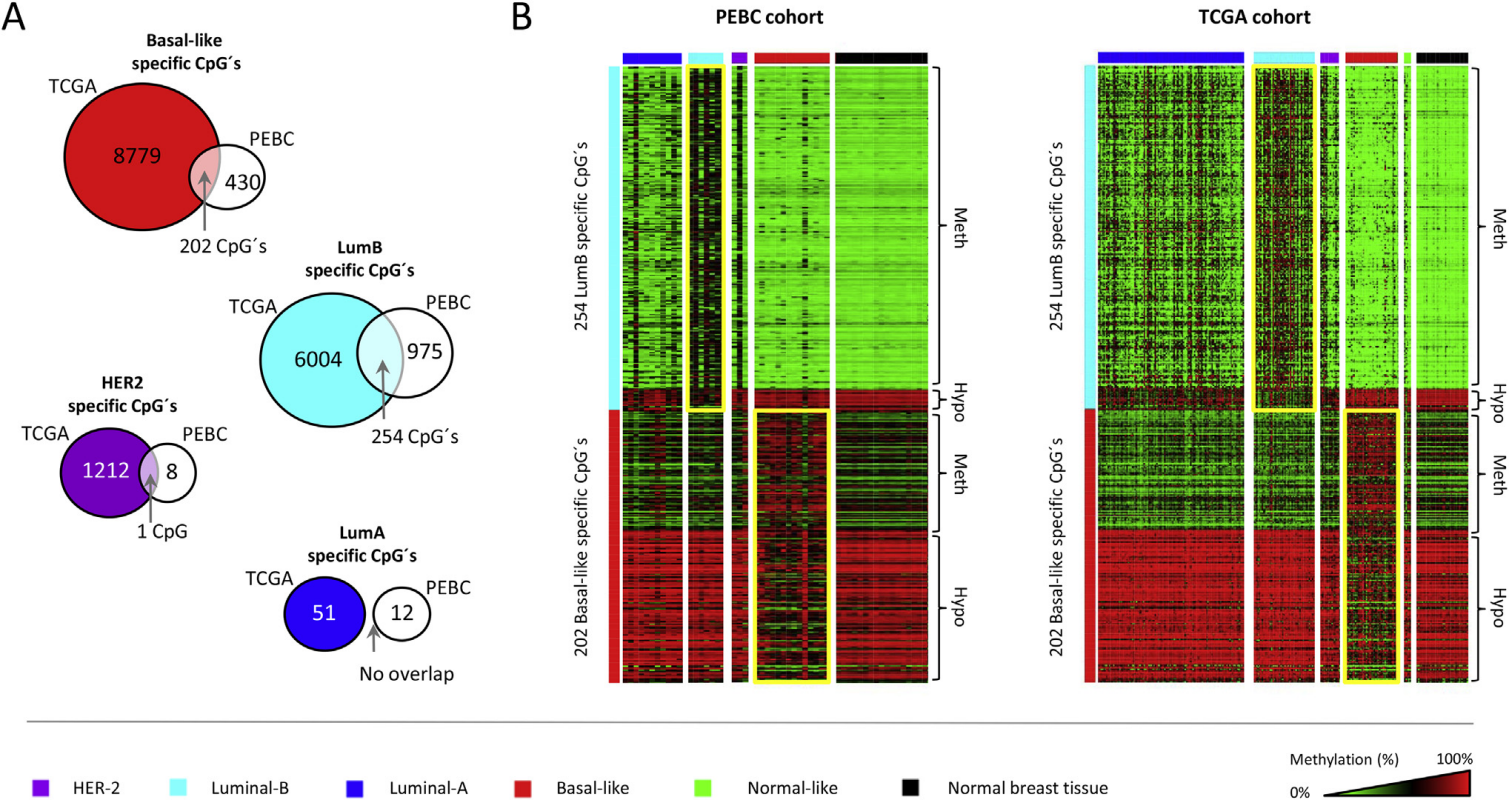
Validation of CpG methylation patterns associated with breast cancer subtypes. A) The subtype‐specific CpG methylation changes identified in relation to each of the four breast cancer subtypes (LumA, LumB, HER2 and Basal‐like) were validated in an independent cohort obtained through the Cancer Genome Atlas. The overlap, i.e. the number of CpG's consistently associated with each of the subtypes in both the TCGA and PEBC cohorts, is indicated by an arrow. B) The validated set of 254 LumB and 202 Basal‐like specific CpG's shown in both cohorts, i.e. the PEBC cohort (left) and the TCGA cohort (right). The expression‐based subtypes are shown on top of the heat‐maps according to the color scheme displayed at the bottom of the figure. Note the heterogeneity among LumB breast tumors, i.e. although most LumB tumors appear to robustly display the LumB‐methylation pattern there are still those that do not and, furthermore, some of the tumors classified as either LumA or HER2 by expression analysis appear to display the LumB‐associated CpG methylation pattern. The CpG methylation states identified as methylated or hypomethylated (relative to normal breast tissue samples) are indicated on the right hand side of the heat‐maps. The heat‐map colors reflect beta‐values representing the degree of methylation from low to high as green to red, respectively (wherein black represents heterogenous/hemi‐methylation), as shown on the scale at the bottom‐right hand side of the figure.

Given the observed potential of DNA methylation changes for identifying biologically aggressive breast cancer subtypes, notably those identified as LumB and Basal‐like (as revealed through the cluster analysis), we derived methylation signatures for each subtype through a multi‐class procedure (uni‐variate) with permutations to adjust for multiple hypothesis testing (Figure 1B; Supplementary Figure 2). The subtype‐specific CpG's identified through this procedure were validated against an independent cohort (The Cancer Genome Atlas; TCGA) wherein breast cancer subtypes have been annotated for each tumor by the PAM50 assay using expression arrays (as described in Parker et al., 2009). The overlap between CpG's identified as specific for the expression‐based subtypes in our cohort (PEBC) and the TCGA cohort is shown in Figure 2A. This analysis revealed consistent changes specific for breast cancers of the LumB and Basal‐like subtypes with 254 and 202 CpG's found specific for LumB and Basal‐like breast cancers, respectively, in both cohorts (Figure 2A; Supplementary Table 2). In contrast, breast cancers of the LumA and HER2 subtypes showed very limited or no overlap at all (Figure 2A). The validated set of subtype‐specific CpG's are displayed in Figure 2B. Here, we note that while most LumB tumors appear to robustly display the methylation pattern associated with the LumB subype, a subset of LumB tumors do not and, furthermore, an appreciable number of LumA and HER2 tumors appear to display LumB‐methylation characteristics (Figure 2B). In contrast, the Basal‐like specific CpG methylation pattern appears specific for basal‐like tumors ‐ especially when looking at the hypomethylated CpG's (Figure 2B). Nonetheless, it is clear that some basal‐like tumors do not conform to the basal‐like methylation pattern, i.e. a few basal‐like tumors appear to be “out of place” (Figure 2B). These observations are addressed and extended further in Section 3.3.

**Figure 3:**
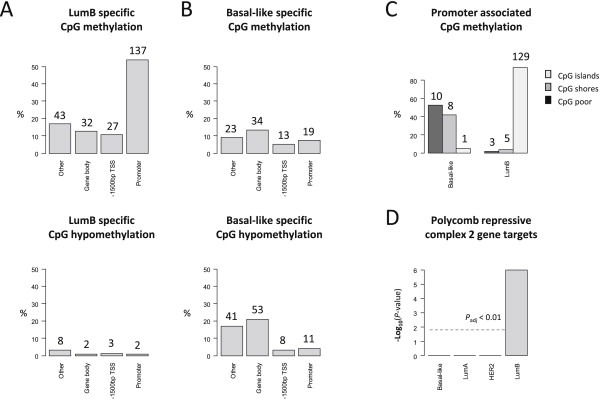
CpG sequence characteristics of the identified subtype‐specific methylation changes. A) The validated DNA methylation signatures specific for LumB (254 CpG's) and B) Basal‐like (202 CpG's) breast cancers differ significantly with respect to the sequence context in which CpG methylation changes tend to occur. The *P*‐values corresponding to a chi‐squared contingency tests indicate “hallmark” features statistically significant for each of the two subtypes involving promoter methylation events for the LumB subtype (*χ* ^2^ = 14.7; *P*‐value = 0.002) and gene body hypomethylation for the Basal‐like subtype (*χ* ^2^ = 9.8; *P*‐value = 0.02). The percentages are computed over all subtype‐specific CpG's wherein the total number was 254 CpG's for the LumB subtype and 202 CpG's for the Basal‐like subtype (with the number of CpG's given for each count; on the top of each bar). The categories analyzed include 1) gene promoter regions, 2) ‐1500 bp TSS indicative of CpG sites located between 1500 bp and 200 bp upstream of transcription start sites (TSS); thus representing potential cis regulatory regions without involving the promoter region and 3) the gene body sites representing sequences located within the gene body. Lastly, the “other” category predominantly represents CpG's found in intergenic regions, i.e. those found outside of the cis regulatory regions (i.e. those not included in the categories of either promoter or ‐1500 bp CpG's) while also not included in the gene body category. C) Promoters displaying methylation in association with either the Basal‐like or LumB subtypes analyzed in terms of CpG islands, CpG shores or CpG poor promoter regions. The percentages are computed for each subtype separately (i.e. Basal‐like and LumB) based on the total number of promoter methylation events specific for each of the two subtypes, i.e. a total of 19 CpG's for the Basal‐like subtype and 137 CpG's for the LumB subtype. D) The genes affected by promoter methylation within the validated subtype‐specific signatures analyzed in terms of whether or not they have been identified as targets of the Polycomb group repressor complex 2 (PRC2). The *P*‐values were derived from Fisher's exact hypothesis testing and the threshold line indicated (grey line) reflects the *P*_adjusted_ < 0.01 significance level after Bonferroni adjustment for multiple hypothesis testing.

The resulting DNA methylation signatures for LumB and Basal‐like tumors, based on the validated catalogue of subtype‐specific CpG's (i.e. the overlapping 254 LumB specific CpG's and 202 Basal‐like specific CpG's), were analyzed further in terms of functionally relevant DNA sequence elements. This analysis revealed that the LumB signature predominantly involves CpG methylation of promoter sequences (54%, 137 of 254; see Figure 3A) whereas the Basal‐like signature predominantly involved hypomethylation events occurring in gene body regions (26%, 53 of 202; see Figure 3B). As this analysis only includes the catalogue of validated CpG's between the two cohorts (i.e. the overlap between subtype‐specific CpG's identified in both the PEBC and TCGA cohorts), the LumA and HER2 subtypes were excluded due to the lack of consistently associated CpG's (see further in Figure 2A).

In exploring events involving CpG promoter methylation further, i.e. focusing only on promoter regions, we found that promoter methylation events within the LumB signature almost exclusively involve CpG islands rather than shores (Figure 3C). In contrast, Basal‐like breast cancers are more or less equally likely to involve CpG shores and other CpG poor promoter regions (Figure 3C). We further demonstrate that the 90 genes uniquely affected by promoter methylation over the 137 CpG's consistently identified in association with the LumB subtype (see Supplementary Table 2) are significantly enriched as targets of the Polycomb group repressor complex 2 (PCR2) in embryonic stem cells (*P*_adjusted_ < 0.01; Figure 3D). Specifically, 31 out of these 90 genes (31 of 90; 34.4%) are known targets of the PCR2 complex. This fraction (34.4%) is significantly higher than can be expected by chance assuming that the list of known PCR2 target genes encompasses 652 genes (*P*_adjusted_ < 0.01; Figure 3D; see also Supplementary Methods). The same was not true for Basal‐like breast cancers regardless of whether we based the analysis on the catalogue of 19 validated promoter associated CpG's (i.e. the catalogue of 19 promoter methylation linked CpG's associated with Basal‐like tumors in both PEBC & TCGA cohorts), or the catalogue of “non‐validated” promoter associated CpG's (i.e. those identified as Basal‐like specific events in either the PEBC or TCGA cohorts separately; data not shown).

### 3.3 DNA methylation‐based definition of breast cancer subtypes

**Figure 4:**
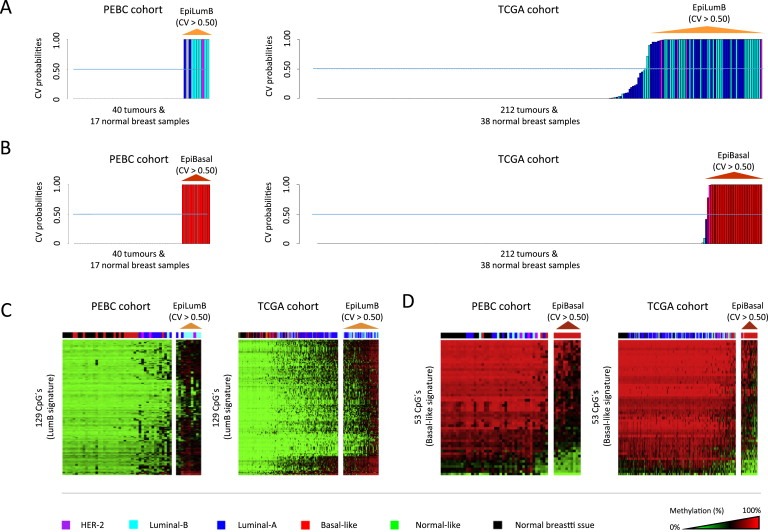
The definition of DNA methylation‐based subtypes in breast tumors. A) Cross‐validated probability values derived from a well‐established pattern recognition algorithm (PAMr implemented in R) indicating how robustly each tumor displays the validated signature of LumB‐associated CpG island promoter methylation events (based on the validated catalogue of LumB‐associated CpG island promoter methylation events, i.e. the 129 CpG's indicated in Figure 3C). The barplots show the probability values on the y‐axis for each tumor ordered on the x‐axis from left to right according to the probability values from low to high, respectively, for each of the two cohorts i.e. the PEBC cohort (barplot on the left) and the TCGA cohort (barplot on the right). Tumors scoring positive for this signature have a cross‐validated probability >0.50 as indicated by the dashed line over each of the two barplots. The color of each bar (representing tumors) is indicative of the expression‐based subtype as given at the bottom of the figure. B) The cross‐validated probability values derived from PAMr indicating how robustly each tumor displays the Basal‐like associated gene body hypomethylation signature (based on the 53 CpG's as indicated in the lower panel of Figure 3B) shown for both the PEBC (left) and TCGA cohorts (right). C) DNA methylation data over the validated catalogue of 129 “hallmark” CpG's characteristic of LumB tumors (i.e. those identified within CpG island promoters in association with the LumB subtype consistently in both the PEBC and TCGA cohorts) shown with respect to the novel Epi‐LumB subtype. The expression‐based subtypes are shown according to the color scheme displayed at the bottom of the figure. D) Similarly, the DNA methylation data over the validated catalogue of 53 “hallmark” CpG's characteristic of Basal‐like tumors (i.e. gene body CpG's consistently associated with Basal‐like tumors in both the PEBC and TCGA cohorts) are shown with respect to the novel Epi‐Basal subtype.

Given the distinctive epigenomic features observed in association with LumB and Basal‐like breast cancers, i.e. CpG island promoter methylation and gene body hypomethylation in LumB and Basal‐like tumors, respectively, we set out to determine how unique these “hallmark features” are to each of the two expression‐based subtypes. This was carried out using a well‐established algorithm for pattern recognition, by which we determined the “degree of similarity” (reflected in the cross‐validation probabilities) for each tumor to the signatures of LumB‐associated CpG island promoter methylation (consisting of 129 CpG's as shown in Figure 3C) and Basal‐like associated gene body hypomethylation (consisting of 53 CpG's as shown in the lower panel of Figure 3B). In this analysis, we found that the signature of LumB‐associated CpG island promoter methylation events effectively characterized most, but not all, of the LumB tumors (6 of 7; 85.7%) while also characterizing two tumors of the LumA (2 of 11; 18.2%) and one of the HER2 subtype (1 of 3; 33.3%) (Figure 4A). In summary, out of the 40 tumors analyzed in our cohort, 10 tumors (10 of 40; 25%) robustly displayed the LumB‐signature. Similar results were obtained based on the analysis of the TCGA cohort, wherein we find that the majority but not all of the LumB tumors robustly display the LumB‐associated CpG island promoter methylation signature (33 of 46; 71.7%) (Figure 4A). While, at the same time, a few members of the LumA (29 of 108; 26.8%) and HER2‐enriched subtype (4 of 14; 28.6%) also display the LumB‐signature. In summary, out of the 212 tumors analyzed in the TCGA cohort, 66 tumors (66 of 212; 31.1%) robustly displayed the LumB‐associated signature of CpG island promoter methylation events. The presence of LumB‐linked methylome characteristics in an appreciable proportion of LumA and HER2 associated tumors provides the basis for defining a novel subtype hereafter referred to as Epi‐LumB (the “Epi” prefix indicating its epigenetic nature).

In looking at the gene body hypomethylation signature associated with the Basal‐like subtype, we found that most (though not all), of the basal‐like tumors in our cohort robustly display this signature (11 of 14; 78.6%) (Figure 4B). In summary, we find no members of any other subtype displaying the basal‐like associated gene body hypomethylation signature in our cohort (the PEBC cohort). Thus, out of the 40 tumors analyzed in the PEBC cohort, 11 displayed this basal‐like signature (11 of 40; 27.5%). In the TCGA cohort, we similarly find that most but not all of the basal‐like tumors (30 of 39; 76.9%) robustly display the basal‐like associated gene body hypomethylation signature. In this cohort, only one member of another subtype displayed this signature, i.e. a HER2‐enriched tumor (1 of 14; 7.1%). Thus, to summarize, out of the 212 tumors analyzed in the TCGA cohort, a total of 31 robustly displayed the basal‐like gene body hypomethylation signature (31 of 212; 14.6%). Given the unique methylome characteristics, i.e. gene body hypomethylation, we refer to the group of tumors that robustly display the gene body hypomethylation signature as the Epi‐Basal subtype. The CpG methylation signatures leading to the classification of tumors as either Epi‐LumB or Epi‐Basal are shown in Figure 4C and D, respectively.

### 3.4 Gene promoter methylation events affecting known cancer genes found in association with the DNA methylation‐based subtypes

We found that the inherent propensity of Epi‐LumB tumors to acquire CpG island promoter methylation events affected a subset of previously established tumor suppressor genes (based on the catalogue of 716 TSG, see Zhao et al., 2013) of which at least five are shown to be strongly down‐regulated upon CpG promoter methylation, i.e. *L3MBTL4*, *ID4*, *IRX1*, *PTCH2* and *RASSF10* (Table 1). The Epi‐Basal subtype, in contrast, was not found to be associated with CpG methylation over the promoter region of known tumor suppressor genes. We note, however, that the analysis of both cohorts supports the observation that basal‐like breast cancers, regardless of whether or not they are Epi‐Basal, significantly associate with promoter methylation of the *BRCA1* gene ‐ a central tumor suppressor gene in breast cancer (data no shown). *BRCA1* promoter methylation, however, does not hold significantly associated with the Epi‐Basal subtype (data not shown). This event, i.e. *BRCA1* promoter methylation, is therefore likely to be more specifically related to the basal‐like phenotype rather than the gene body hypomethylation phenotype.

**Table 1:** Epi‐LumB specific CpG methylation events (significant at the <1% FDR in both the PEBC and TCGA cohorts) were found to affect a subset of previously known tumor suppressor genes (based on the catalogue of 716 TSG's). The statistics shown describe the relation between CpG methylation and expression over Epi‐LumB‐associated TSG's in the TCGA cohort where data was available on both CpG methylation and expression (by RNA sequencing) for 731 tumors and 82 normal breast tissue samples. Statistically significant hits are shown (including a minimum threshold of R2 > 0.10 and fold change > 2).

TargetID	Gene symbol	R2	Fold change in expression (Unmethylated/Methylated)	P‐value (adjusted)
cg14352983	L3MBTL4	0.264	2.667	4.96E‐55
cg08336641	L3MBTL4	0.259	3.004	1.12E‐53
cg14155416	L3MBTL4	0.255	2.621	7.90E‐53
cg12924825	L3MBTL4	0.253	2.902	2.17E‐52
cg18556788	L3MBTL4	0.245	2.652	2.02E‐50
cg17688525	L3MBTL4	0.241	2.710	1.43E‐49
cg03715143	ID4	0.238	2.850	8.89E‐49
cg09232937	IRX1	0.191	6.137	2.08E‐38
cg05724871	L3MBTL4	0.177	2.625	1.99E‐35
cg14271531	ID4	0.147	2.676	5.32E‐29
cg21167628	PTCH2	0.116	2.028	7.70E‐23
cg20918243	RASSF10	0.109	9.006	2.33E‐21
cg10530883	IRX1	0.106	5.251	8.22E‐21

### 3.5 Distinct tumor evolutionary paths in association with Epi‐LumB and Epi‐Basal tumors

**Figure 5:**
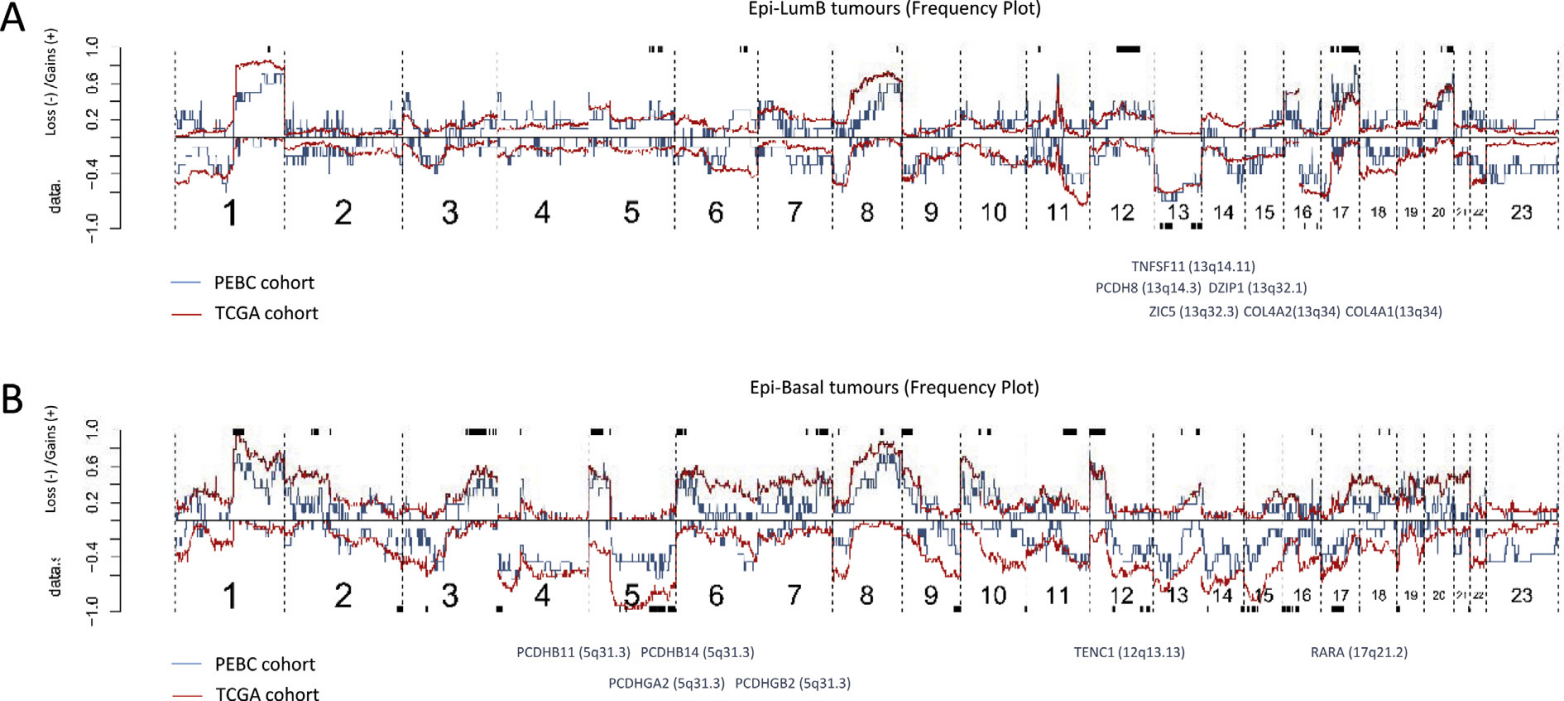
Patterns of DNA copy number changes associated with Epi‐LumB and Epi‐Basal tumors revealing divergent tumor evolutionary paths and candidate tumor suppressor genes. A) Frequencies of DNA copy number gains and losses in Epi‐LumB tumors are displayed as proportions on the y‐axis (positive and negative signs represent gains and losses, respectively) according to genomic position along the x‐axis from p‐arm to q‐arm (left to right, respectively) for all 23 chromosomes. The blue and red lines are frequencies derived from the PEBC and TCGA cohorts, respectively. The bars (black) on top and below the frequency plot indicate positions wherein copy number gains and losses, respectively, were found to be associated with Epi‐LumB tumors (comparing Epi‐LumB against all other tumors). Only regions wherein the statistical significance achieved less than 5% FDR in both the PEBC and TCGA cohorts are shown. A subset of the genes found within significant regions of copy number loss were also found to be promoter methylated in association with the Epi‐LumB subtype. The identity of these candidate tumor suppressor genes are given below the frequency plot. B) Event frequency plot for Epi‐Basal tumors (upper), with the corresponding significance analysis (black bars above and below the frequency plot indicative of significant copy number gains and losses, respectivley). Epi‐Basal associated promoter methylation events (gene symbols) found within significant regions of copy number losses are shown.

The different patterns, or signatures, of changes in CpG methylation states are indicative of divergent evolutionary paths. To test this hypothesis, we studied the patterns of DNA copy number changes in the same cohort of breast tumors as those analyzed using the 450K Infinium methylation method. Here, we identified highly recurrent copy number changes that were significantly associated with Epi‐LumB tumors in both cohorts involving deletions over chromosomes 13q and 16p, together with copy number gains of chromosomes 12q, 17q and 20q (Figure 5A). In this analysis, we identified subtype‐specific events for Epi‐LumB tumors involving either DNA copy number loss or CpG promoter methylation over *DZIP1*, *TNFSF11*, *ZIC5*, *COL4A2*, *COL4A1* and *PCDH8* indicating candidate tumor suppressor genes (Figure 5A; Supplementary Figure 3). Of these genes, *DZIP1* and *COL4A2* show a significant relation between promoter methylation and down‐regulated expression based on data from the TCGA cohort (data not shown).

As expected, given the overlap with the basal‐like subtype, we found that tumors of the Epi‐Basal subtype displayed highly aberrational landscapes of DNA copy number changes. Many of the DNA copy number changes observed in Epi‐Basal tumors were recurrent such as deletions over 4p, 5q, 12q and 17p as well as copy number gains over 7q, 9p and 12p (Figure 5B). Here, subtype‐specific events involving either DNA copy number loss or CpG promoter methylation were found to involve *TENC1*, *RARA*, *PCDHB11*, *PCDHB14*, *PCDHGA2* and *PCDHGB2* indicative of candidate tumor suppressor genes in this context (Figure 5B; Supplementary Figure 3). Based on the TCGA cohort, the *TENC1*, *PCDHB11* and *PCDHGA2* genes show a clear relation between promoter methylation and down‐regulated expression (data not shown).

### 3.6 The clinical relevance of DNA methylation‐based subtypes

To determine the clinical relevance of the DNA methylation‐based subtypes (the Epi‐LumB and Epi‐Basal subtypes), we developed locus‐specific assays for analyzing a few selected markers that could serve as proxies for tumor classification. We selected *TTBK1*, *ZNF132 and KCNA3* from the catalogue of top significant CpG island promoter methylation events (only taking into account CpG's located in gene promoters containing CpG islands) associated with Epi‐LumB tumors (Supplementary Table 3). In designing an assay for the Epi‐Basal subtype, there are a number of problems that arise with detecting gene body hypomethylation including the identification of a region that is recurrently hypomethylated while, at the same time, the change from high to low levels of methylation (in normal tissue compared with tumors, respectively), would need to be substantial to enable accurate and sensitive detection in clinical samples. The presence of stromal cells, the normal breast epithelial tissue and lymphocytes in such samples all add to the difficulties with this type of analysis. Although these factors were not an obstacle in discovering the gene body hypomethylation phenotype (i.e. in relation to the Epi‐Basal subtype), see Supplementary Figure 4, they can lead to unreliable results in the clinical setting. For these reasons we selected CpG promoter methylation events, rather than body hypomethylation events, identified as top significant markers in association with tumors classified as Epi‐Basal. As this assay does not directly measure the phenotype of interest, i.e. gene body hypomethylation, it should be understood that it may simply reflect another way for classifying basal‐like breast cancers (an alternative to expression analyses) – especially since nearly all of the basal‐like breast cancers included in the discovery cohort were found to display the gene body hypomethylation phenotype as revealed in Figure 4B and D. Regardless of this, it is of interest to determine whether a DNA methylation‐based assay for the Epi‐Basal subtype can improve the prognostic value for this subtype contrasted against the expression‐based definition alone. Based on this reasoning, we selected *ZNF671* and *TENC1* promoter methylation as putative markers of Epi‐Basal breast cancers from the top promoter methylation events identified as significantly associated with tumors classified as Epi‐Basal (Supplementary Table 3). Although the selection of subtype‐specific methylation markers was based on the top significant CpG's indicative of different breast cancer subtypes (not taking into account their effects on gene expression), we find that the proxy markers *KCNA3*, *ZNF132*, *TENC1* and *ZNF671* are down‐regulated in association with promoter methylation based on RNA sequencing and methylation data from the TCGA cohort (Supplementary Figure 5).

**Figure 6:**
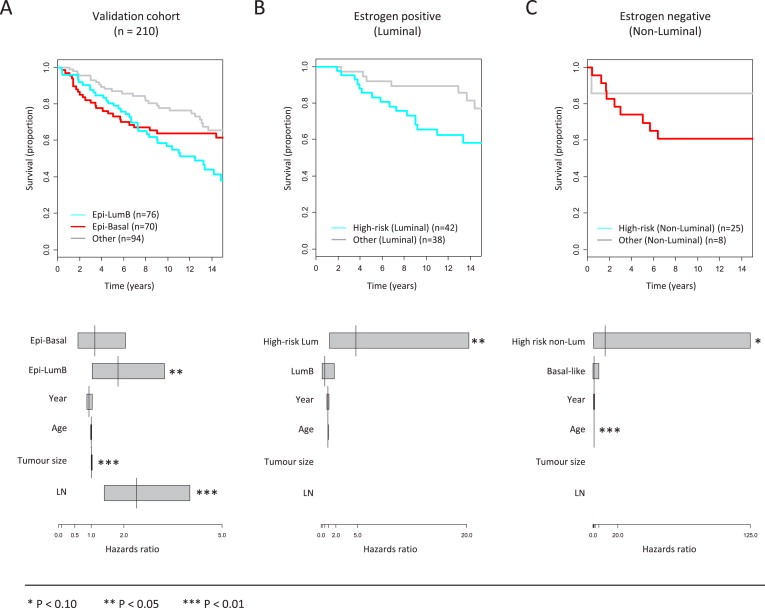
Breast cancer‐specific survival analysis with respect to the novel DNA methylation defined subtypes, i.e. the Epi‐LumB and Epi‐Basal subtypes. A) Patients with breast tumors classified as either Epi‐LumB or Epi‐Basal on the basis of proxy CpG methylation markers (see Section 3.6 for details) are associated with reduced time to death due to breast cancer (i.e. breast cancer‐specific death). The lower panel displays a multivariate Cox's proportional hazards modeling of the survival data wherein the Epi‐LumB subtype was found to be an independent prognostic factor after adjustment for tumor size, lymph node metastases and age‐ and year at diagnosis. B) In analyzing a subset of the tumors wherein information on hormone receptor status (along with subtype‐specific markers) was available, we show that ER positive tumors classified as either Epi‐LumB (based on proxy‐methylation markers) or LumB (based on the Ki‐67 expression marker) represents an improvement above the LumB definition alone for predicting reduced time to breast cancer‐specific death. The lower panel shows the multivariate Cox's proportional hazards regression model for ER positive tumors classified as either Epi‐LumB (based on the proxy‐methylation markers) or LumB (based on high Ki‐67 expression) referred to as “high‐risk luminal tumors”, demonstrating the superior prognostic value in combining epigenetic and expression data above that of expression data alone. C) The Epi‐Basal subtype combined with the basal‐like expression‐based definition (using EGFR and CK5/6) was not significantly associated with reduced time to breast cancer‐specific deaths in ER negative breast cancers. However, as shown in the lower panel, the combination of epigenetic and expression data provides superior prognostic value of marginal statistical significance above that of expression data alone. Here, ER negative tumors classified as either Epi‐Basal (on the basis of proxy‐methylation markers) or basal‐like (based on expression of either EGFR or CK5/6), referred to as “high‐risk non‐luminal tumors” remain marginally significant for predicting breast cancer‐specific deaths while the basal‐like definition alone does not hold significant.

Pyrosequencing was then used to analyze the selected proxy markers for Epi‐LumB and Epi‐Basal tumors in an independent validation cohort of primary breast tumor samples from 310 patients. The Epi‐LumB subtype was then assigned to tumors displaying methylation over the promoter region of two out of the three surrogate markers (*TTBK1*, *ZNF132* and *KCNA3*). The Epi‐Basal subtype was then assigned to tumors negative for the Epi‐LumB phenotype while positive for methylation of either *TENC1* or *ZNF671*. In support for the validity of this classification system, we find that LumB tumors are enriched within the Epi‐LumB subtype while basal‐like tumors are enriched within the Epi‐Basal subtype (Table 2). The Epi‐LumB and Epi‐Basal subtypes, defined according to this proxy‐based classification system, were both found to be significantly associated with greater tumor size and poorly differentiated phenotypes (Table 3). Importantly, the results revealed significantly shorter survival times for patients that develop Epi‐LumB subtype breast tumors after adjustment for tumor size, the presence of lymph node metastases along with age and year at diagnosis (Hazards‐ratio = 1.83; *P* = 0.035) (Figure 6A).

**Table 2:** DNA methylation defined subtypes in an independent cohort validating the relation to the classification of breast cancers according to expression‐based subtypes.a

	Basal‐like	HER2	LumA	LumB	5NPb	Total
Epi‐LumB	2 (8%)	2 (8%)	7 (29%)	11 (46%)	2 (8%)	24 (100%)
Epi‐Basal	10 (40%)	1 (4%)	6 (24%)	7 (28%)	1 (4%)	25 (100%)
Other	0	0	25 (62%)	14 (35%)	1 (3%)	40 (100%)
						X 2 = 31.0; P = 0.00014

a Information on expression‐based subtype classification was available in 89 of the tumors included in the validation cohort.

b 5NP represents unclassified tumors due to negativity for the five phenotypic markers ER, PR, HER2, CK5/6 and EGFR.

**Table 3:** The clinical relevance of Epi‐LumB and Epi‐Basal tumors, defined according to selected proxy markers, analyzed with respect to parameters of clinical staging (tumor size and nodal metastasis status) and degree of differentiation (histological grade).

Tumor size	T1a‐c	T2 – T3	Total
Epi‐LumB	18 (26%)	52 (74%)	68 (100%)
Epi‐Basal	17 (30%)	40 (70%)	56 (100%)
Other	46 (52%)	42 (48%)	88 (100%)
			X 2 = 13.7; P = 0.0010
Nodal metastases	Negative	Positive	Total
Epi‐LumB	23 (35%)	43 (65%)	64 (100%)
Epi‐Basal	19 (36%)	34 (64%)	52 (100%)
Other	37 (49%)	38 (51%)	75 (100%)
			X 2 = 3.8; P = 0.15
Histological grading	1+/2+	3+	Total
Epi‐LumB	12 (32%)	25 (67%)	35 (100%)
Epi‐Basal	11 (32%)	23 (68%)	33 (100%)
Other	47 (78%)	13 (22%)	54 (100%)
			X 2 = 27.6; P < 0.0001

### 3.7 Improved identification of highly aggressive breast cancers by combining methylation‐ and expression‐based subtype definitions

We find that breast cancer‐specific survival times in LumB breast cancer patients do not differ depending on whether or not the tumors are positive for the Epi‐LumB phenotype (data not shown). Similarly, survival of patients with basal‐like breast cancers does not differ depending on whether or not the tumors are positive for the Epi‐Basal phenotype (data not shown). These results indicate that the prognostic value associated with methylation‐ and expression‐based breast cancer subtype definitions do not differ significantly – although we note that the number of patients in the independent cohort with available information on both definitions entails limited statistical power.

Based on these results, we addressed the question of whether methylation markers can improve the prognostic value associated with standard expression‐based definition of breast cancer subtypes. This was carried out using multivariate Cox proportional hazards regression analyses in estrogen positive and negative disease separately to then test whether the addition of the Epi‐LumB and Epi‐Basal subtype definitions, respectively, would provide additional information on disease prognosis beyond that provided by expression‐based markers alone. Using this approach, we first focused exclusively on estrogen‐receptor (ER) positive disease and show that ER + tumors displaying either the LumB expression‐based phenotype or positivity for the Epi‐LumB markers (grouped together as “high‐risk luminal”) have approximately 5‐fold increased risk for breast cancer‐specific death (Hazards ratio = 4.63; *P* = 0.035) and, importantly, this effect was independent of the LumB expression‐based phenotype alone in a model allowing adjustment for age‐ and year at diagnosis (Figure 6B). In estrogen‐receptor negative disease, the low number of ER‐tumors did not reveal formal statistical significance (Figure 6C). However, we note that a trend can be observed wherein ER‐tumors that are positive for either the basal‐like expression markers (CK5/6 or EGFR positivity) or the Epi‐Basal markers (grouped together as “high‐risk non‐luminal”) are marginally associated with approximately 10‐fold increased risk for breast cancer‐specific death independently of the expression‐based definition for basal‐like cancers alone in a model with adjustment for age and year at diagnosis (Hazards ratio = 9.79; *P* = 0.079) (Figure 6C, lower panel).

## 4 Discussion

In this report we identify DNA methylation signatures that are associated with biologically distinct breast cancer subtypes. We show that the signatures identified are characterized not only by differences in the catalogue of genes affected but also in the CpG context, i.e. in the DNA sequences that tend to undergo changes in methylation states. This involves a significantly higher propensity for CpG island methylation at promoter sequences in breast tumors of the Luminal‐B subtype (LumB), whereas gene body hypomethylation events characterize those of the Basal‐like subtype. This suggests that the mechanisms predominantly responsible for changes in the landscape of breast cancer methylomes differ between biologically distinct cancer subtypes. Based on these results, we explored the clinical relevance of defining novel DNA methylation‐based subtypes according to the hallmark features associated with each of the two identified signatures. To address this, we developed locus‐specific assays for selected proxy markers to identify 1) the signature of LumB‐associated CpG island promoter methylation events defining the Epi‐LumB subtype, and 2) the signature of Basal‐like associated gene body hypomethylation events defining the Epi‐Basal subtype. The analysis of these selected proxy markers in a larger independent cohort of patients reveals rapid disease progression in the context of poorly differentiated phenotypes in tumors classified as either Epi‐LumB or Epi‐Basal. The clinical importance of this finding relates to the identification of patients that will require adjuvant treatment options and close follow‐up. The failure to identify patients with aggressive disease can result in sub‐optimal outcomes and, possibly, patient death that could otherwise have been avoided or substantially delayed. In turn, it is well established that a considerable fraction of patients are being unnecessarily treated with cytotoxic drugs. The novel DNA methylation‐defined subtypes described here could therefore be helpful as prognostic parameters for making decision regarding the treatment of breast cancer patients.

It has previously been shown that CpG island promoter methylation events in cancers tend to affect genes regulated by the Polycomb repressor group complex 2 (PRC2) marked for repression by histone modifications involving the H3K27Me3 mark in embryonic stem cells (Viré et al., 2006). Our results support this finding and demonstrate that this property characterizes the majority of breast cancers classified as LumB. In contrast, we find that the tendency to display CpG island promoter methylation events does not specifically characterize Basal‐like tumors. These tumors, however, are preferentially characterized by gene body hypomethylation events suggesting that a fundamentally different mechanism is effectively more responsible for driving epigenetic changes in breast cancers of the Basal‐like subtype. The gene bodies of actively transcribed genes are generally found heavily methylated (Jones, 2012). The functional relevance of gene body hypomethylation events is still unclear, however, a recent paper describes hypomethylated gene bodies as a characteristic of repressed genes in a cancer cell line model (Hon et al., 2012). Further support for a causative relation between hypomethylation of gene bodies and loss of expression has been reported by other research groups (Yang et al., 2014; Varley et al., 2013). Thus, loss of gene body methylation described here as a hallmark feature of Basal‐like tumors could represent a mechanism of gene silencing.

By using data on DNA copy number changes, we identified genes of potential interest as tumor suppressors in the development of Epi‐LumB and Epi‐Basal tumors, including *DZIP1* and *COL4A2* (Epi‐LumB) and *TENC1* (Epi‐Basal). In fact, CpG promoter methylation of the *TENC1* gene ranked among the top scoring genes as a proxy marker for the Epi‐Basal subtype. The *TENC1* gene (officially named tensin like C1 domain containing phosphatase tensin 2) has not previously been identified as a tumor suppressor gene although it has been shown to physically interact with *DLC1* gene products (a well‐established tumor suppressor gene in liver cancer) through which it contributes to growth suppression (Chan et al., 2009). Likewise, none of the Epi‐LumB specific markers, i.e. *ZNF132*, *TTBK1* and *KCNA3* have been described as tumor suppressors and it is not clear at this point whether epigenetic inactivation of these genes represent driver events that contribute to cancer development. Indeed, the possibility remains that CpG island promoter methylation of these genes may simply reflect the outcome of processes taking place on a genome‐wide scale. As an example, it has been suggested that loss of protective barriers guarding CpG islands against cytosine methylation are responsible for CpG island promoter methylation in cancer; in which case PRC2‐regulated genes can simply be seen as more vulnerable than other genes for CpG island promoter methylation as they are already targeted for repression in normal cells. In this scenario, the vast majority of CpG island promoter methylated genes represent passenger events with only a few true cancer driving events found in each case; in this context, *DZIP1*, *COL4A2*, *L3MBTL4*, *IRX1* and *RASSF10* are named as likely candidates. Nonetheless, CpG island promoter methylation of the three Epi‐LumB proxy markers identified here (*ZNF132*, *TTBK1* and *KCNA3*) are more consistently observed across all tumors of this subtype and thus more appropriate as proxies for identifying tumors of this subtype regardless of whether they represent true cancer driving events or not.

The causative factors implicated in the methylator phenotype are currently emerging with exciting advances taking place in gliomas following the identification of acquired mutations in the *IDH1* gene (Noushmehr et al., 2010). *IDH1* mutations were found to be correlated with high frequency of CpG island methylation events in gliomas and subsequently shown to be a causative factor in this regard (Noushmehr et al., 2010; Turcan et al., 2012). More recently, the value of *IDH1* inhibitors has now been established in pre‐clinical models as a possible anti‐cancer drug specifically active in *IDH1* mutated cancer cells leading to delayed growth and the induction of differentiation (Rohle et al., 2013). However, the *IDH1* gene is rarely mutated in cancers other than gliomas and is therefore not a likely candidate as a causative factor for the CpG methylator phenotype in breast cancers (Kandoth et al., 2013). Nevertheless, recent efforts aimed at cancer genome sequencing have revealed recurrent mutations in epigenetic genes, i.e. genes involved in regulating chromatin dynamics and the processing of epigenetic marks (Stephens et al., 2012; Cancer Genome Atlas Network, 2012). Examples include mutations in the *ARID1A* gene occurring in a subset of ovarian cancers (Jones et al., 2010) and breast cancers (Mamo et al., 2012) along with mutations in the *MLL2*, *MLL3*, *SMARCD1*, *NCOR1* and *ARID1B* genes found in a subset of breast cancers (Stephens et al., 2012; Cancer Genome Atlas Network, 2012; Kandoth et al., 2013). The distinct DNA methylation signatures we describe can be a great complement to the recently described genetic and expression (Stephens et al., 2012; Curtis et al., 2012; Dvinge et al., 2013) profiles to translate genomic analyses to clinical applications in breast cancer.

## 5 Conclusions

Breast cancer is a heterogenous disease with different subtypes showing distinct biological and clinical features. An important problem in breast cancer treatment is the definition of patient subsets that will require aggressive treatment options and close follow‐up after treatment. By studying the DNA methylation landscape of breast cancers, we have discovered signatures associated with two biologically distinct and aggressive breast cancer subtypes known as Basal‐like and Luminal‐B. The identified signatures underlie the definition of two novel DNA methylation‐based subtypes referred to here as Epi‐Basal and Epi‐LumB, respectively. We show that the DNA methylation defined subtypes hold prognostic value that could provide helpful information beyond that of other clinical parameters for making decisions on whether or not cytotoxic therapy will be necessary. In this context, we show that only a few selected proxy markers are sufficient to classify tumors according to DNA methylation‐based subtypes using a cost‐effective method and therefore easily incorporated into the clinic.

## Financial support

The Icelandic Centre for Research RANNIS (J.E., H.H.), the Cellex Foundation (ME), Fundación Sandra Ibarra de Solidaridad frente al Cáncer (M.E.), Junta de Barcelona of the Asociación Española Contra el Cáncer (O.A.S.), and the Health and Science Departments of the Catalan Government (Generalitat de Catalunya) (M.E.). M.E. is an ICREA Research Professor.

## Conflicts of interest

The authors declare no conflicts of interests.

## Supporting information



Supplementary data

## Supplementary data 1

### 1.1

Supplementary data related to this article can be found at http://dx.doi.org/10.1016/j.molonc.2014.10.012.

## References

[mol2201593555-bib-0001] Bediaga, N.G. , Acha-Sagredo, A. , Guerra, I. , Viguri, A. , Albaina, C. , Ruiz Diaz, I. , Rezola, R. , Alberdi, M.J. , Dopazo, J. , Montaner, D. , Renobales, M. , Fernández, A.F. , Field, J.K. , Fraga, M.F. , Liloglou, T. , de Pancorbo, M.M. , 2010 DNA methylation epigenotypes in breast cancer molecular subtypes. Breast Cancer Res. 12, (5) R77 20920229 10.1186/bcr2721 PMC3096970

[mol2201593555-bib-0002] Cancer Genome Atlas Network , 2012 Comprehensive molecular portraits of human breast tumors. Nature. 490, (7418) 61–70. 23000897 10.1038/nature11412 PMC3465532

[mol2201593555-bib-0003] Chan, L.K. , Ko, F.C. , Ng, I.O. , Yam, J.W. , 2009 Deleted in liver cancer 1 (DLC1) utilizes a novel binding site for Tensin2 PTB domain interaction and is required for tumor-suppressive function. PLoS One. 4, (5) e5572 19440389 10.1371/journal.pone.0005572 PMC2680019

[mol2201593555-bib-0004] Chin, K. , DeVries, S. , Fridlyand, J. , Spellman, P.T. , Roydasgupta, R. , Kuo, W.L. , Lapuk, A. , Neve, R.M. , Qian, Z. , Ryder, T. , Chen, F. , Feiler, H. , Tokuyasu, T. , Kingsley, C. , Dairkee, S. , Meng, Z. , Chew, K. , Pinkel, D. , Jain, A. , Ljung, B.M. , Esserman, L. , Albertson, D.G. , Waldman, F.M. , Gray, J.W. , 2006 Genomic and transcriptional aberrations linked to breast cancer pathophysiologies. Cancer Cell. 10, (6) 529–541. 17157792 10.1016/j.ccr.2006.10.009

[mol2201593555-bib-0005] Curtis, C. , Shah, S.P. , Chin, S.F. , Turashvili, G. , Rueda, O.M. , Dunning, M.J. , Speed, D. , Lynch, A.G. , Samarajiwa, S. , Yuan, Y. , Gräf, S. , Ha, G. , Haffari, G. , Bashashati, A. , Russell, R. , McKinney, S. , METABRIC Group , Langerød, A. , Green, A. , Provenzano, E. , Wishart, G. , Pinder, S. , Watson, P. , Markowetz, F. , Murphy, L. , Ellis, I. , Purushotham, A. , Børresen-Dale, A.L. , Brenton, J.D. , Tavaré, S. , Caldas, C. , Aparicio, S. , 2012 The genomic and transcriptomic architecture of 2,000 breast tumors reveals novel subgroups. Nature. 486, (7403) 346–352. 22522925 10.1038/nature10983 PMC3440846

[mol2201593555-bib-0006] Dedeurwaerder, S. , Desmedt, C. , Calonne, E. , Singhal, S.K. , Haibe-Kains, B. , Defrance, M. , Michiels, S. , Volkmar, M. , Deplus, R. , Luciani, J. , Lallemand, F. , Larsimont, D. , Toussaint, J. , Haussy, S. , Rothé, F. , Rouas, G. , Metzger, O. , Majjaj, S. , Saini, K. , Putmans, P. , Hames, G. , van Baren, N. , Coulie, P.G. , Piccart, M. , Sotiriou, C. , Fuks, F. , 2011 DNA methylation profiling reveals a predominant immune component in breast cancers. EMBO Mol. Med. 3, (12) 726–741. 21910250 10.1002/emmm.201100801 PMC3377115

[mol2201593555-bib-0007] Diaz-Cruz, E.S. , Cabrera, M.C. , Nakles, R. , Rutstein, B.H. , Furth, P.A. , 2010 BRCA1 deficient mouse models to study pathogenesis and therapy of triple negative breast cancer. Breast Dis. 32, (1–2) 85–97. 21778574 10.3233/BD-2010-0308 PMC3500619

[mol2201593555-bib-0008] Dvinge, H. , Git, A. , Gräf, S. , Salmon-Divon, M. , Curtis, C. , Sottoriva, A. , Zhao, Y. , Hirst, M. , Armisen, J. , Miska, E.A. , Chin, S.F. , Provenzano, E. , Turashvili, G. , Green, A. , Ellis, I. , Aparicio, S. , Caldas, C. , 2013 The shaping and functional consequences of the microRNA landscape in breast cancer. Nature. 497, (7449) 378–382. 23644459 10.1038/nature12108

[mol2201593555-bib-0009] Fang, F. , Turcan, S. , Rimner, A. , Kaufman, A. , Giri, D. , Morris, L.G. , Shen, R. , Seshan, V. , Mo, Q. , Heguy, A. , Baylin, S.B. , Ahuja, N. , Viale, A. , Massague, J. , Norton, L. , Vahdat, L.T. , Moynahan, M.E. , Chan, T.A. , 2011 Breast cancer methylomes establish an epigenomic foundation for metastasis. Sci. Transl. Med. 3, (75) 75ra25 10.1126/scitranslmed.3001875 PMC3146366 21430268

[mol2201593555-bib-0010] Heyn, H. , Esteller, M. , 2012 DNA methylation profiling in the clinic: applications and challenges. Nat. Rev. Genet. 13, (10) 679–692. 22945394 10.1038/nrg3270

[mol2201593555-bib-0011] Holm, K. , Hegardt, C. , Staaf, J. , Vallon-Christersson, J. , Jönsson, G. , Olsson, H. , Borg, A. , Ringnér, M. , 2010 Molecular subtypes of breast cancer are associated with characteristic DNA methylation patterns. Breast Cancer Res. 12, (3) R36 20565864 10.1186/bcr2590 PMC2917031

[mol2201593555-bib-0012] Hon, G.C. , Hawkins, R.D. , Caballero, O.L. , Lo, C. , Lister, R. , Pelizzola, M. , Valsesia, A. , Ye, Z. , Kuan, S. , Edsall, L.E. , Camargo, A.A. , Stevenson, B.J. , Ecker, J.R. , Bafna, V. , Strausberg, R.L. , Simpson, A.J. , Ren, B. , 2012 Global DNA hypomethylation coupled to repressive chromatin domain formation and gene silencing in breast cancer. Genome Res. 22, (2) 246–258. 22156296 10.1101/gr.125872.111 PMC3266032

[mol2201593555-bib-0013] Hortobagyi, G.N. , de la Garza Salazar, J. , Pritchard, K. , Amadori, D. , Haidinger, R. , Hudis, C.A. , Khaled, H. , Liu, M.C. , Martin, M. , Namer, M. , O'Shaughnessy, J.A. , Shen, Z.Z. , Albain, K.S. , ABREAST Investigators , 2005 The global breast cancer burden: variations in epidemiology and survival. Clin. Breast Cancer. 6, (5) 391–401. 16381622 10.3816/cbc.2005.n.043

[mol2201593555-bib-0014] Jones, P.A. , 2012 Functions of DNA methylation: islands, start sites, gene bodies and beyond. Nat. Rev. Genet. 13, (7) 484–492. 22641018 10.1038/nrg3230

[mol2201593555-bib-0015] Jones, S. , Wang, T.L. , Shih, IeM. , Mao, T.L. , Nakayama, K. , Roden, R. , Glas, R. , Slamon, D. , Diaz, L.A. , Vogelstein, B. , Kinzler, K.W. , Velculescu, V.E. , Papadopoulos, N. , 2010 Frequent mutations of chromatin remodeling gene ARID1A in ovarian clear cell carcinoma. Science. 330, (6001) 228–231. 20826764 10.1126/science.1196333 PMC3076894

[mol2201593555-bib-0016] Kandoth, C. , McLellan, M.D. , Vandin, F. , Ye, K. , Niu, B. , Lu, C. , Xie, M. , Zhang, Q. , McMichael, J.F. , Wyczalkowski, M.A. , Leiserson, M.D. , Miller, C.A. , Welch, J.S. , Walter, M.J. , Wendl, M.C. , Ley, T.J. , Wilson, R.K. , Raphael, B.J. , Ding, L. , 2013 17 Mutational landscape and significance across 12 major cancer types. Nature. 502, (7471) 333–339. 24132290 10.1038/nature12634 PMC3927368

[mol2201593555-bib-0017] López-Tarruella, S. , Martín, M. , 2009 Recent advances in systemic therapy. Advances in adjuvant systemic chemotherapy of early breast cancer. Breast Cancer Res. 11, 204 19344489 10.1186/bcr2226 PMC2688937

[mol2201593555-bib-0018] Mamo, A. , Cavallone, L. , Tuzmen, S. , Chabot, C. , Ferrario, C. , Hassan, S. , Edgren, H. , Kallioniemi, O. , Aleynikova, O. , Przybytkowski, E. , Malcolm, K. , Mousses, S. , Tonin, P.N. , Basik, M. , 2012 An integrated genomic approach identifies ARID1A as a candidate tumor-suppressor gene in breast cancer. Oncogene. 31, (16) 2090–2100. 21892209 10.1038/onc.2011.386

[mol2201593555-bib-0019] Noushmehr, H. , Weisenberger, D.J. , Diefes, K. , Phillips, H.S. , Pujara, K. , Berman, B.P. , Pan, F. , Pelloski, C.E. , Sulman, E.P. , Bhat, K.P. , Verhaak, R.G. , Hoadley, K.A. , Hayes, D.N. , Perou, C.M. , Schmidt, H.K. , Ding, L. , Wilson, R.K. , Van Den Berg, D. , Shen, H. , Bengtsson, H. , Neuvial, P. , Cope, L.M. , Buckley, J. , Herman, J.G. , Baylin, S.B. , Laird, P.W. , Aldape, K. , Cancer Genome Atlas Research Network , 2010 Identification of a CpG island methylator phenotype that defines a distinct subgroup of glioma. Cancer Cell. 17, (5) 510–522. 20399149 10.1016/j.ccr.2010.03.017 PMC2872684

[mol2201593555-bib-0020] Parker, J.S. , Mullins, M. , Cheang, M.C. , Leung, S. , Voduc, D. , Vickery, T. , Davies, S. , Fauron, C. , He, X. , Hu, Z. , Quackenbush, J.F. , Stijleman, I.J. , Palazzo, J. , Marron, J.S. , Nobel, A.B. , Mardis, E. , Nielsen, T.O. , Ellis, M.J. , Perou, C.M. , Bernard, P.S. , 2009 Supervised risk predictor of breast cancer based on intrinsic subtypes. J. Clin. Oncol. 27, (8) 1160–1167. 19204204 10.1200/JCO.2008.18.1370 PMC2667820

[mol2201593555-bib-0021] Perou, C.M. , Børresen-Dale, A.L. , 2011 Systems biology and genomics of breast cancer. Cold Spring Harb. Perspect. Biol. 3, (2) 10.1101/cshperspect.a003293 PMC3039533 21047916

[mol2201593555-bib-0022] Rohle, D. , Popovici-Muller, J. , Palaskas, N. , Turcan, S. , Grommes, C. , Campos, C. , Tsoi, J. , Clark, O. , Oldrini, B. , Komisopoulou, E. , Kunii, K. , Pedraza, A. , Schalm, S. , Silverman, L. , Miller, A. , Wang, F. , Yang, H. , Chen, Y. , Kernytsky, A. , Rosenblum, M.K. , Liu, W. , Biller, S.A. , Su, S.M. , Brennan, C.W. , Chan, T.A. , Graeber, T.G. , Yen, K.E. , Mellinghoff, I.K. , 2013 An inhibitor of mutant IDH1 delays growth and promotes differentiation of glioma cells. Science. 340, (6132) 626–630. 23558169 10.1126/science.1236062 PMC3985613

[mol2201593555-bib-0023] Saal, L.H. , Gruvberger-Saal, S.K. , Persson, C. , Lövgren, K. , Jumppanen, M. , Staaf, J. , Jönsson, G. , Pires, M.M. , Maurer, M. , Holm, K. , Koujak, S. , Subramaniyam, S. , Vallon-Christersson, J. , Olsson, H. , Su, T. , Memeo, L. , Ludwig, T. , Ethier, S.P. , Krogh, M. , Szabolcs, M. , Murty, V.V. , Isola, J. , Hibshoosh, H. , Parsons, R. , Borg, A. , 2008 Recurrent gross mutations of the PTEN tumor suppressor gene in breast cancers with deficient DSB repair. Nat. Genet. 40, (1) 102–107. 18066063 10.1038/ng.2007.39 PMC3018354

[mol2201593555-bib-0024] Sigurdardottir, L.G. , Jonasson, J.G. , Stefansdottir, S. , Jonsdottir, A. , Olafsdottir, G.H. , Olafsdottir, E.J. , Tryggvadottir, L. , 2012 Data quality at the Icelandic Cancer Registry: comparability, validity, timeliness and completeness. Acta Oncol. 51, (7) 880–889. 22974093 10.3109/0284186X.2012.698751

[mol2201593555-bib-0025] Sorlie, T. , Tibshirani, R. , Parker, J. , Hastie, T. , Marron, J.S. , Nobel, A. , Deng, S. , Johnsen, H. , Pesich, R. , Geisler, S. , Demeter, J. , Perou, C.M. , Lønning, P.E. , Brown, P.O. , Børresen-Dale, A.L. , Botstein, D. , 2003 Repeated observation of breast tumor subtypes in independent gene expression data sets. Proc. Natl. Acad. Sci. U S A. 100, (14) 8418–8423. 12829800 10.1073/pnas.0932692100 PMC166244

[mol2201593555-bib-0026] Stefansson, O.A. , Esteller, M. , 2013 Epigenetic modifications in breast cancer and their role in personalized medicine. Am. J. Pathol. 183, (4) 1052–1063. 23899662 10.1016/j.ajpath.2013.04.033

[mol2201593555-bib-0027] Stephens, P.J. , Tarpey, P.S. , Davies, H. , Van Loo, P. , Greenman, C. , Wedge, D.C. , Nik-Zainal, S. , Martin, S. , Varela, I. , Bignell, G.R. , Yates, L.R. , Papaemmanuil, E. , Beare, D. , Butler, A. , Cheverton, A. , Gamble, J. , Hinton, J. , Jia, M. , Jayakumar, A. , Jones, D. , Latimer, C. , Lau, K.W. , McLaren, S. , McBride, D.J. , Menzies, A. , Mudie, L. , Raine, K. , Rad, R. , Chapman, M.S. , Teague, J. , Easton, D. , Langerød, A. , Oslo Breast Cancer Consortium (OSBREAC) , Lee, M.T. , Shen, C.Y. , Tee, B.T. , Huimin, B.W. , Broeks, A. , Vargas, A.C. , Turashvili, G. , Martens, J. , Fatima, A. , Miron, P. , Chin, S.F. , Thomas, G. , Boyault, S. , Mariani, O. , Lakhani, S.R. , van de Vijver, M. , van 't Veer, L. , Foekens, J. , Desmedt, C. , Sotiriou, C. , Tutt, A. , Caldas, C. , Reis-Filho, J.S. , Aparicio, S.A. , Salomon, A.V. , Børresen-Dale, A.L. , Richardson, A.L. , Campbell, P.J. , Futreal, P.A. , Stratton, M.R. , 2012 The landscape of cancer genes and mutational processes in breast cancer. Nature. 486, (7403) 400–404. 22722201 10.1038/nature11017 PMC3428862

[mol2201593555-bib-0028] Tibshirani, R. , Hastie, T. , Narasimhan, B. , Chu, G. , 2002 Diagnosis of multiple cancer types by shrunken centroids of gene expression. Proc. Natl. Acad. Sci. U S A. 99, (10) 6567–6572. 12011421 10.1073/pnas.082099299 PMC124443

[mol2201593555-bib-0029] Turcan, S. , Rohle, D. , Goenka, A. , Walsh, L.A. , Fang, F. , Yilmaz, E. , Campos, C. , Fabius, A.W. , Lu, C. , Ward, P.S. , Thompson, C.B. , Kaufman, A. , Guryanova, O. , Levine, R. , Heguy, A. , Viale, A. , Morris, L.G. , Huse, J.T. , Mellinghoff, I.K. , Chan, T.A. , 2012 IDH1 mutation is sufficient to establish the glioma hypermethylator phenotype. Nature. 483, (7390) 479–483. 22343889 10.1038/nature10866 PMC3351699

[mol2201593555-bib-0030] Varley, K.E. , Gertz, J. , Bowling, K.M. , Parker, S.L. , Reddy, T.E. , Pauli-Behn, F. , Cross, M.K. , Williams, B.A. , Stamatoyannopoulos, J.A. , Crawford, G.E. , Absher, D.M. , Wold, B.J. , Myers, R.M. , 2013 Dynamic DNA methylation across diverse human cell lines and tissues. Genome Res. 23, (3) 555–567. 23325432 10.1101/gr.147942.112 PMC3589544

[mol2201593555-bib-0031] Viré, E. , Brenner, C. , Deplus, R. , Blanchon, L. , Fraga, M. , Didelot, C. , Morey, L. , Van Eynde, A. , Bernard, D. , Vanderwinden, J.M. , Bollen, M. , Esteller, M. , Di Croce, L. , de Launoit, Y. , Fuks, F. , 2006 The polycomb group protein EZH2 directly controls DNA methylation. Nature. 439, (7078) 871–874. 16357870 10.1038/nature04431

[mol2201593555-bib-0032] Yang, X. , Han, H. , De Carvalho, D.D. , Lay, F.D. , Jones, P.A. , Liang, G. , 2014 Gene body methylation can alter gene expression and is a therapeutic target in cancer. Cancer Cell. 26, (4) 577–590. pii: S1535-6108(14)00316-X 25263941 10.1016/j.ccr.2014.07.028 PMC4224113

[mol2201593555-bib-0033] Zhao, M. , Sun, J. , Zhao, Z. , 2013 TSGene: a web resource for tumor suppressor genes. Nucleic Acids Res. 41, (Database issue) D970–D976. 23066107 10.1093/nar/gks937 PMC3531050

