# Supplementary material for: A DNA methylation‐based definition of biologically distinct breast cancer subtypes
Source: Mol Oncol. 2014 Nov 5;9(3):555–68. doi: 10.1016/j.molonc.2014.10.012 (PMC5528700; doi:10.1016/j.molonc.2014.10.012)
Supplement: Supplementary file 1 — Supplementary data [file MOL2-9-555-s001.docx]

**Necessary Additional Data**

***Stefansson OA, Moran S, et al. A DNA methylation-based definition of biologically distinct breast cancer subtypes*.**

**Inventory:**

**Supplementary Methods**

**Supplementary Figures 1, 2, 3, 4 and 5**

**Supplementary Tables 1, 2,and 3**

**Supplementary Methods**

**Infinium HumanMethylation450 BeadChips**

High-quality DNA samples obtained from tumors (n=40) and normal breast tissue (n=17) were selected for bisulfite conversion (Zymo Research; EZ-96 DNA Methylation™ Kit) and hybridization to Infinium HumanMethylation450 BeadChips (Illumina) following Illumina’s Infinium HD Methylation protocol. The quality control sample standards were DNA concentration measurements by the PicoGreen method (Invitrogen) coupled with assessment of DNA purity based on A260/A280 ratios (ranging between 1·75 and 1·95) and A260/A230 ratios (ranging between 2·00 and 2·20). Analysis by 1% agarose gel permitted exclusion of samples with possible DNA fragmentation or RNA contamination. The Infinium HumanMethylation450 BeadChip provides coverage of > 450,000 CpG sites targeting nearly all RefSeq genes (> 99%).^1^ The chips were designed to cover coding and non-coding genes without bias against those lacking CpG islands. The design further aimed to cover not only promoter regulatory regions, but also CpGs across gene regions in order to include 5'-untranslated regions (5´UTRs), the first exons, the gene bodies and 3´-untranslated regions (3´UTRs). Approximately 96% of CpG islands were covered along with regions proximal to the CpG islands (“CpG shores”) and, more distal, CpG shelves. A total of 600 ng high-quality DNA samples were bisulfite converted. Whole-genome amplification and hybridization were then carried out on the BeadChip, followed by single-base extension and analysis on the HiScan SQ module (Illumina) to assess cytosine methylation states.

**Pyrosequencing**

The most significant markers within the signature defining the Epi-LumB subtype were selected as proxies (TTBK1, KCNA3and ZNF132) (Supplementary Table 3). In designing assays for the Epi-Basal subtype, several problems arise when detecting gene body hypomethylation, including the identification of a region that is recurrently hypomethylated, while the change from high to low levels of methylation in normal tissue compared with tumors would need to be substantial to allow accurate and sensitive detection in clinical samples. The presence of stromal cells, the normal breast epithelial tissue and lymphocytes in such samples all add to the difficulties with this type of analysis. For these reasons, we selected CpG promoter methylation events, rather than hypomethylation, identified within the signature for the Epi-Basal subtype as potential surrogate markers of this phenotype (Supplementary Table 3). In this way, we identified CpG island promoter methylation of the ZNF671 and TENC1 genes as correlated features for the Epi-Basal phenotype (Supplementary Table 3). The PyroMark Q96 system (Qiagen) was used to carry out pyrosequencing of bisulfite-converted DNA. The EZ-96 DNA Methylation-Gold™ Kit (Zymo Research) was used for bisulfite conversion. The pyrosequencing method enables quantification of the degree of methylation over selected CpG sites. The primer sequences used in this analysis were designed using Qiagen’s Pyromark Assay Design 2·0 software. Five markers were sequenced: CpG island promoter methylation of TTBK1, ZNF132, KCNA3, ZNF671 and TENC1. The primer sequences used were (all given 5´ > ´3): TTBK1 prePCR fwd: [Btn]ATGTTAGGGTTGGTGTTGA, TTBK1 pre-PCR reverse: CCCCRCCTCACCTACTCTATACC, TTBK1 seq: ATAACCCRCTCCCTCC. ZNF132 prePCR fwd: AGAGGTTTGTTGTAGTTTAATTTATTAGT, ZNF132 prePCR rev: [Btn]ATAAACCCCATTCCTCAATCACCTTAAC, ZNF132 seq: GTTTTTATTGGTTTAAGGATTTT. ZNF671 prePCR fwd: GGAGTYGGAGAAAGGGTGATTGA, ZNF671 prePCR rev:

[Btn]CATTTTATTTCTATCAAACTATCCCTAACC, ZNF671 seq: GGGGAYGTAGGTATT. TENC1 prePCR fwd: [Btn]TTAGGTTGGAGTTTGGTTGGGATAT, TENC1 prePCR rev: CCAACTCTAAAATTATCCCACACCT, TENC1 seq: CCATAATCTTAAAATTCTACCC. KCNA3 prePCR fwd: [Btn]GGTAGYGGTGAGGTTAGGT, KCNA3 prePCR rev: AACCRACTCTACTAAATAAACATTCC, KCNA3 prePCR seq: ACTACAAAAAATACAAACCC. The PrePCR was carried out using IMMOLASE (Bioline, USA) hot-start DNA polymerase in a touchdown PCR reaction (96°C 10 min; 45 cycles of {98°C for 30 s, 61°C annealing temperature for 30 s w/-0.2°C each cycle, 72°C for 30 s} end of cycling; 72°C for 15 min). For quality control, at least five samples were run on agarose gels to validate the presence of a single sharp band of the expected size with no extra background-amplified products present. Pyro Q-CpG software (Qiagen) was used to assess the degree of methylation, expressed as percentages from 0% (no methylation detected) to 100% (all sequenced CpGs detected as methylated). The definition of promoter methylation by pyrosequencing was based on the analysis of the 25 normal breast samples (derived from the normal breast adjacent to the site of tumorous tissue) with three standard deviations above the mean of each marker (mean + 3*SD). This criteria gives the following marker-specific thresholds: KCNA3 (mean=3.54%; SD=4.68; KCNA3-threshold > 20%), ZNF132 (mean=1.99%; SD=3.17; ZNF132-threshold > 10%), TTBK1 (mean=3.37; SD=1.85, TTBK1-threshold > 10%), TENC1 (mean=14.7%; SD=4.12; TENC1-threshold > 30%), ZNF671 (mean=2.0; SD=2.72; ZNF671-threshold > 10%). Cases of potential errors due to incomplete bisulfite conversion or low signal peaks, detected by the Pyro Q-CpG software, were discarded. The sequencing of the promoter regions of TTBK1, ZNF132 and ZNF671 was successful in 325, 287 and 279 of these tumors, respectively (performed in both discovery and validation cohorts, i.e., all 350 tumors in the study).

**DNA copy number analysis and tissue microarrays**

DNA copy number changes were analyzed by aCGH (array comparative genomic hybridization) using 385K oligonucleotide aCGH microarrays (Roche NimbleGen, Inc., Reykjavik, Iceland) as previously reported.^2^ This design contains 385,000 probes covering the human genome with ~1 probe for each 7000 bp. DNA was labeled using Cy3- and Cy5 for DNA derived from tumor and normal blood samples from the same patients, respectively, followed by hybridization on microarrays according to protocols developed by the manufacturer (NimbleGen Arrays User’s Guide-CGH Analysis, Roche NimbleGen, Inc., Reykjavik, Iceland). Tissue microarrays (TMAs) used in this study were constructed beforehand and analyzed for the expression of subtype-specific markers, i.e., ER, PR, HER-2, Ki-67, EGFR and CK5/6, by immunohistochemistry (IHC).^3^ Based on this analysis, tumors were assigned to breast cancer subtypes according to a validated classification scheme.^4,5,6^ Tumors positive for either ER or PR were classified as Luminal. High levels of expression of Ki-67 (> 14%) in breast tumors with a Luminal phenotype were classified as Luminal-B (LumB), the remainder being classified as Luminal-A (LumA). Tumors negative for both ER and PR while positive for HER2 (IHC score 3+) were classified as HER2 subtype. Positivity for either CK5/6 or EGFR in tumors negative for both ER and PR were classified as Basal-like. Negativity for ER, PR, HER2, CK5/6 and EGFR was designated as 5NP (five negative phenotype).

**TP53 mutation analyses**

PCR amplification and constant denaturation gradient electrophoresis (CDGE) were carried out and mutations were confirmed by direct DNA sequencing. PCR conditions, CDGE and sequencing conditions were as described earlier.^7,8^ All samples were screened for mutations in exons 5 to 8 of the TP53 gene.

**Bioinformatic analyses**

DNA methylation data were normalized using the quantile method implemented in lumi (R 2·12·2).^9^ CpGs differentially methylated between normal and breast tumor tissue samples were identified using the samr method, wherein random permutations are made to adjust for multiple hypothesis testing (samr package in R 2·12·1).^10^ We defined significant associations as those in which the false-discovery rate was < 5%. In exploring the potential of DNA methylation patterns to define significant tumor subtypes/clusters, we selected differentially methylated CpGs showing at least a 10% mean difference (in absoloute beta values, i.e. +/-0.10) between normal and breast tissue samples and a standard deviation of at least 15% within the group of tumor samples. These CpGs were then ranked according to their F score, computed as the difference in average methylation between the normal and tumor samples divided by the ratio between the deviation in normal breast tissue and that in tumor samples. This score is designed to identify CpG sites that differ strongly between samples of normal and tumor tissues while at the same time being highly stable, or similar, within the group of normal breast tissue samples.

In section 3.1, we used the top 5,000 CpG (rankedaccordingto the F-score described above) to construct cluster dendrograms using hierarchical clustering with complete linkage and the Manhattan distance as a measure of similarity (heatmap.2 function implemented in the gplot package for R). By using the pvclust package (R 2·12·1), we defined significant tumor clusters as those showing AU > 90 (at least four cluster members).

In section 3.2. we used the multiclass approach using the samr package for R to identify CpG sites differentially methylated between the different expression-based subtypes (i.e. LumA, LumB, HER2 and Basal-like). This analysis was uni-variate (i.e. not adjusted for histological grade and clinical staging). The normal-like breast cancer subtype given in the TCGA cohort was excluded from this analysis as the biological relevance of this subtype is still debated. Statistically significant CpG´s were then identified based on a false-discovery rate (FDR) within 5%. Subsequently, the mean beta value for each CpG site was computed for each subtype and the differences in means determined for each subtype against each of the others (resulting in three values for each CpG site for each of the four subtypes). A threshold of at least +/- 0.10 in mean differences was applied to call subtype-specific CpGs (the direction of change required to be the same for a given subtype being compared against each of the others).

In section 3.2, we further analyse the identified subtype-specific patterns in terms promoter-associated CpGs defined as those located within 200 bp of the transcription start site, or those within the 5´UTR or first exon, i.e. CpG´s proximal to the TSS (transcription start site) and we therefore performed separate analyses for CpG´s located more than 1500bp away from the TSS (those referred to as TSS1500). We further analysed the patterns in terms of curated gene catalogues with known polycomb group repressor complex 2 (PRC2) occupied genes and those marked for repression by the H3K27Me3 mark (lysine residue 27 of histone 3 being attached with the mark of three methyl groups) were downloaded from the Broad Institute of MIT and Harvard website hosting the gene set enrichment analyzer (http://www.broadinstitute.org/gsea/msigdb/genesets.jsp).

In section 3.3., the signatures specific for each subtype were used asinput for the pamr algorithm (pamr package for R). The signatures used were 1) CpG island promoter methylation events associated with the LumB subtype consisting of 129 CpG´s, see Supplementary Table 2 for a list of CpG methylation events associated with LumB together with information on which of these are located within CpG promoter Islands, and 2) gene body hypomethylation events associated with the Basal-like subtype consisting of 53 CpG´s,see Supplementary Table 2 for a list of CpG hypomethylation events determined as Basal-like specific together with information on which of these are located within gene bodies. This involves classifying each tumor according to a leave-one-out cross-validation procedure carried out separately for each of the two signatures. Here, a “conventional” probability threshold of more than 0·50 was defined as significant for a positive classification. The results are then presented in Figure 4.

In section 3.4, we analysed our results in terms of the tumor suppressor catalogue of 716 genes compiled previously by Zhao and colleagues.^11^ This catalogue was downloaded from the TSGene database; <http://bioinfo.mc.vanderbilt.edu/TSGene/>. The overlap between these 716 TSG´s and the catalogue of Epi-LumB specific promoter methylated genes was determined. In Table 1, we list out the overlapping genes (their promoter CpG´s) that additionally were found to be strongly downregulated by methylation events. The relation between CpG methylation and expression was determined in the TCGA cohort where data was available on both methylation and expression in the same samples; this resulting in 713 primary breast tumours and 82 normal breast tissue samples. The *P*-values were derived from a linear regression model followed by a Benjamini-Hochberg adjustment for multiple hypothesis testing. The significant hits shown in Table 1 fulfill additional thresholding of R^2^ > 0.10 and at least a two-fold change in expression (i.e. median expression in unmethylated tumours / median expression in methylated tumours).

In section 3.5., the aCGH data were pre-processed by obtaining the normalized log_2_ ratios,^12^ represented as means within windows of ten probes (10x data representation), then segmented using the CBS algorithm (DNAcopy package for R).^13^ The segmented profiles of each tumor were obtained by replacing each of the ten-probe representations by the corresponding segment means. Event frequency plots were obtained using a threshold of ± 0·075 for the segmented profiles to obtain counts of DNA copy number gains and deletions at each location and histogram plots using standard graphic functions in R. Differences in DNA copy number changes between tumor groups or subtypes were determined using the segmented profiles as input in samr to adjust for multiple hypothesis testing, significant differences being defined as < 5% the false-discovery rate threshold. Additional criteria for reporting characteristic subtype-specific DNA copy number changes were at least two-fold differences in event frequencies and a minimum of three observed events (to avoid conclusion of spurious relationships). The same approach was used for the TCGA cohort making use of the “nocnv” option (somatic CNV´s excluded) in previously segmented Level 3 data.

In section 3.6, we re-analysed the methylation data (both PEBC and TCGA cohorts) in terms of the novel subtypes, i.e. Epi-LumB and Epi-Basal to identify specific promoter methylation events associated with each of the two subtypes for use as proxy markers. The identification of promoter methylation events associated with the Epi-LumB subtype included only CpG´s found within CpG island promoter regions found unmethylated in normal breast tissues. CpG´s were defined as unmethylated in normal breast tissue if their distribution was found to be within the range of 0 <β< 0·25 (considering only the normal breast tissue samples). The SAMr method was then used to identify significantly associated CpG island promoter methylation events separately for tumours classified as either Epi-LumB or Epi-Basal. For the Epi-LumB subtype we applied an FDR threshold of <1% whereas few events fulfilled this criteria for the Epi-Basal subtype and we therefore applied a more relaxed criteria of < 5% FDR. The same procedure was applied for both our cohort (PEBC) and the TCGA cohort. The number of CpG´s found associated with the Epi-LumB and Epi-Basal subtypes in both the PEBC and TCGA cohorts (the overlap) included 5852 and 68 CpG´s, respectively. To prepare a top gene list, we then further trimmed down this list of CpG´s by selecting only those that achieve a within-subtype recurrence rate of at least 50%, i.e. for a given CpG to be determined valid as a potential proxy marker they must be found methylated in at least half of either the Epi-LumB and Epi-Basaltumours. The list of CpG´s fulfilling this criteria for the Epi-LumB and Epi-Basal subtypes in both the PEBC and TCGA cohorts included 3034 and 61 CpG´s, respectively. The top candidate gene promoters were then determined based on ranking the fold-change output (derived from the samr analysis above) for promoters with at least two CpG´s fulfilling the criteria above (in both cohorts). Supplementary Table 3 shows the top 30 gene promoter regions for each of the two Epi subtypes. Note, only 8 candidate gene promoter regions fulfilled all the criteria for the Epi-Basal subtype. Thus, Supplementary Table 3 comprehensively includes all the candidate proxy markers (involving promoter methylation events) for the Epi-Basal subtype whereas only the top 30 are listed for the Epi-LumB subtype.

**Supplementary References**

1. Sandoval J, Heyn H, Moran S, Serra-Musach J, Pujana MA, Bibikova M, Esteller M. Validation of a DNA methylation microarray for 450,000 CpG sites in the human genome. Epigenetics 2011; 6(6): 692-702.

2. Stefansson OA, Jonasson JG, Johannsson OT, Olafsdottir K, Steinarsdottir M, Valgeirsdottir S, Eyfjord JE. Genomic profiling of breast tumours in relation to BRCA abnormalities and phenotypes. Breast Cancer Res. 2009; 11(4): R47.

3. Stefansson OA, Jonasson JG, Olafsdottir K, Hilmarsdottir H, Olafsdottir G, Esteller M, Johannsson OT, Eyfjord JE. CpG island hypermethylation of BRCA1 and loss of pRb as co-occurring events in basal/triple-negative breast cancer. Epigenetics 2011; 6(5): 638-649.

4. Cheang MC, Chia SK, Voduc D, Gao D, Leung S, Snider J, Watson M, Davies S, Bernard PS, Parker JS, Perou CM, Ellis MJ, Nielsen TO. Ki67 index, HER2 status, and prognosis of patients with luminal B breast cancer. J Natl Cancer Inst. 2009; 101(10): 736-50.

5. Cheang MC, Voduc D, Bajdik C, Leung S, McKinney S, Chia SK, Perou CM, Nielsen TO. Basal-like breast cancer defined by five biomarkers has superior prognostic value than triple-negative phenotype. Clin Cancer Res. 2008; 14(5): 1368-76.

6. Blows FM, Driver KE, Schmidt MK, Broeks A, van Leeuwen FE, Wesseling J, Cheang MC, Gelmon K, Nielsen TO, Blomqvist C, Heikkilä P, Heikkinen T, Nevanlinna H, Akslen LA, Bégin LR, Foulkes WD, Couch FJ, Wang X, Cafourek V, Olson JE, Baglietto L, Giles GG, Severi G, McLean CA, Southey MC, Rakha E, Green AR, Ellis IO, Sherman ME, Lissowska J, Anderson WF, Cox A, Cross SS, Reed MW, Provenzano E, Dawson SJ, Dunning AM, Humphreys M, Easton DF, García-Closas M, Caldas C, Pharoah PD, Huntsman D. Subtyping of breast cancer by immunohistochemistry to investigate a relationship between subtype and short and long term survival: a collaborative analysis of data for 10,159 cases from 12 studies. PLoS Med. 2010; 7(5): e1000279.

7. A L Børresen, E Hovig, B Smith-Sørensen, D Malkin, S Lystad, T I Andersen, J M Nesland, K J. Isselbacher, and S H Friend. Constant denaturant gel electrophoresis as a rapid screening technique for p53 mutations. Proc Natl Acad Sci U S A. 1991; 88(19): 8405-8409.

8. Thorlacius S, Börresen AL, Eyfjörd JE. Somatic p53 mutations in human breast carcinomas in an Icelandic population: a prognostic factor. Cancer Res. 1993; 53(7): 1637-1641.

9. Du P, Kibbe WA, Lin SM. lumi: a pipeline for processing Illumina microarray. Bioinformatics 2008; 24(13): 1547-8.

10. Tusher VG, Tibshirani R, Chu G. Significance analysis of microarrays applied to the ionizing radiation response. Proc Natl Acad Sci U S A. 2001; 98(9): 5116–5121.

11. Zhao M, Sun J, Zhao Z. TSGene: a web resource for tumor suppressor genes. Nucleic Acids Res. 2013; 41(Database issue): D970-6.

12. Workman C, Jensen LJ, Jarmer H, Berka R, Gautier L, Nielser HB, Saxild HH, Nielsen C, Brunak S, Knudsen S. A new non-linear normalization method for reducing variability in DNA microarray experiments. Genome Biol. 2002; 3(9): research0048.

13. Olshen AB, Venkatraman ES, Lucito R, Wigler M. Circular binary segmentation for the analysis of array-based DNA copy number data. Biostatistics 2004; 5(4): 557-72.


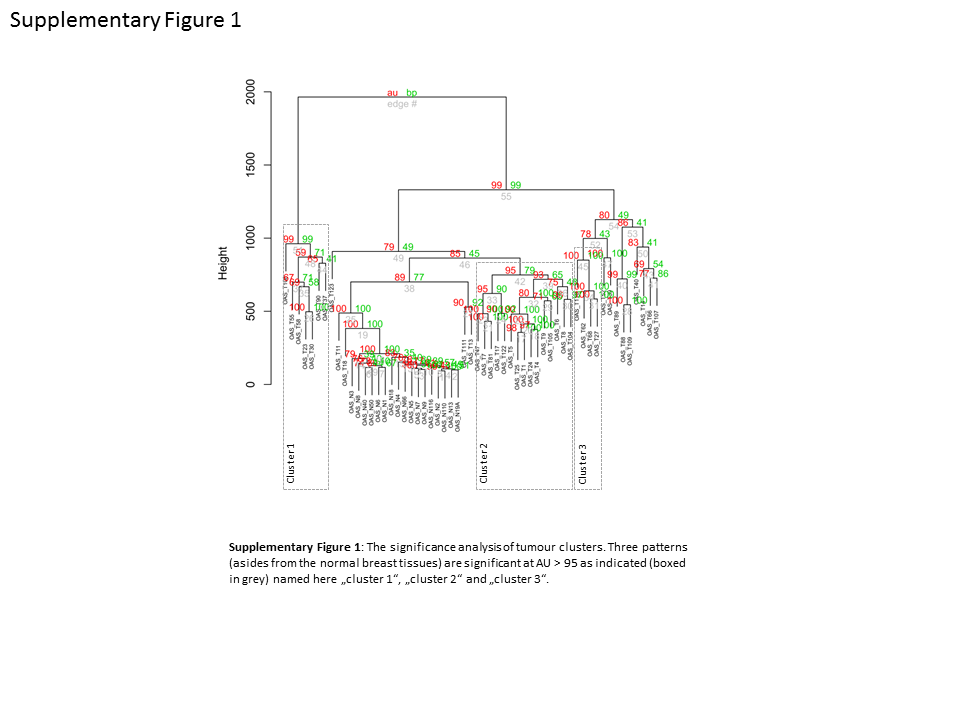


**Supplementary Figure 1:** Significance analysis of tumor clusters (carried out with the pvclust package for R). Three patterns (other than those of normal breast tissues) are significant at AU > 95%, as indicated (boxed in grey): “cluster 1”, “cluster 2” and “cluster 3”.


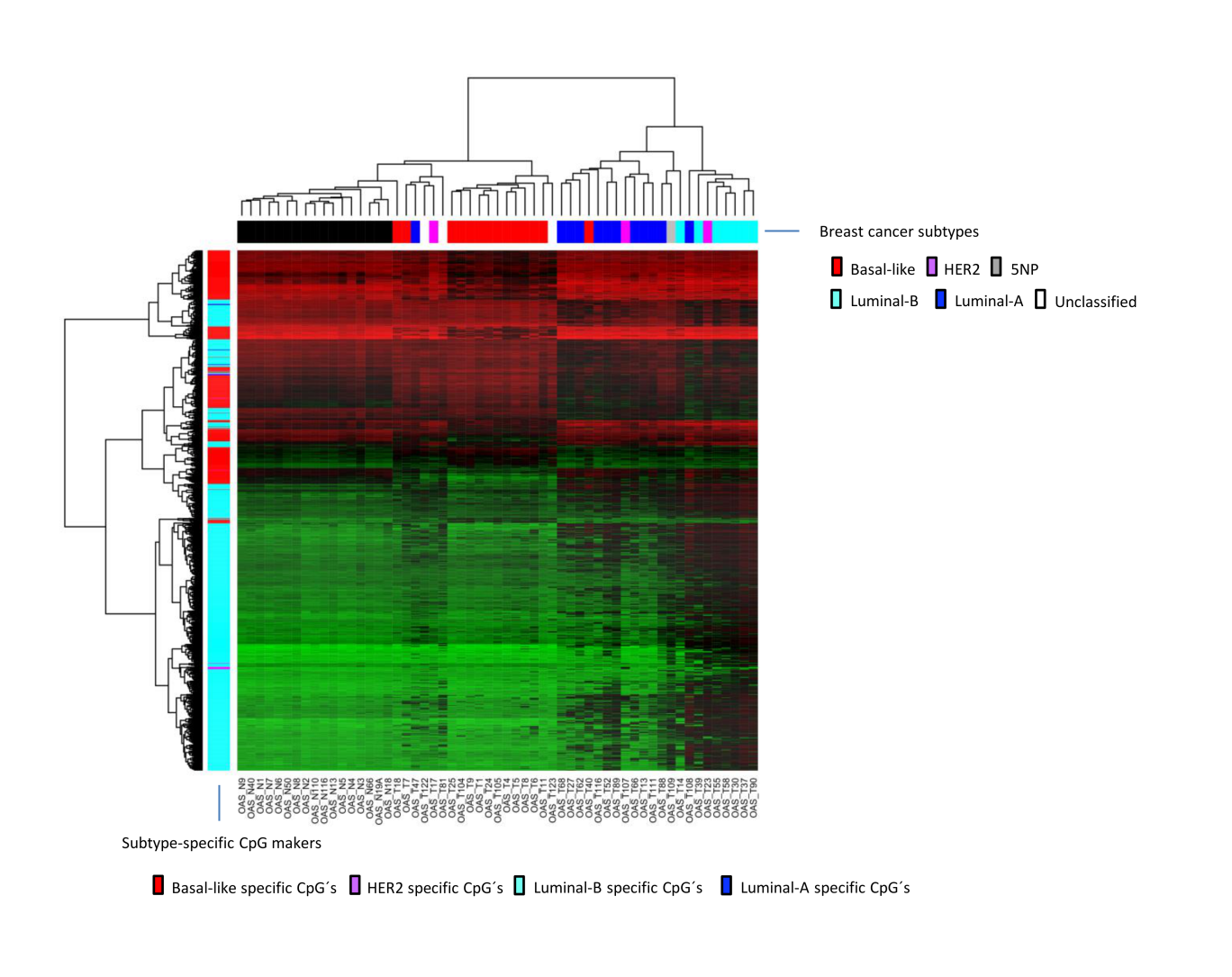


**Supplementary Figure 2:** Hierarchical clustering of all the 1425 CpG´s identified as subtype-specific in the PEBC cohort. The CpG markers (rows) are displayed according to their subtype-specific association in colours as indicated at the bottom of the figure. In summary, this includes 430 CpG´s uniquely associated with the Basal-like subtype, 975 CpG´s uniquely associated with the LumB subtype, 8 CpG´s uniquely associated with the HER2 subtype and 12 CpG´s uniquely associated with the LumA subtype. The subtype class is shown for each sample (columns) as indicated in colors at the upper-right hand side of the figure.


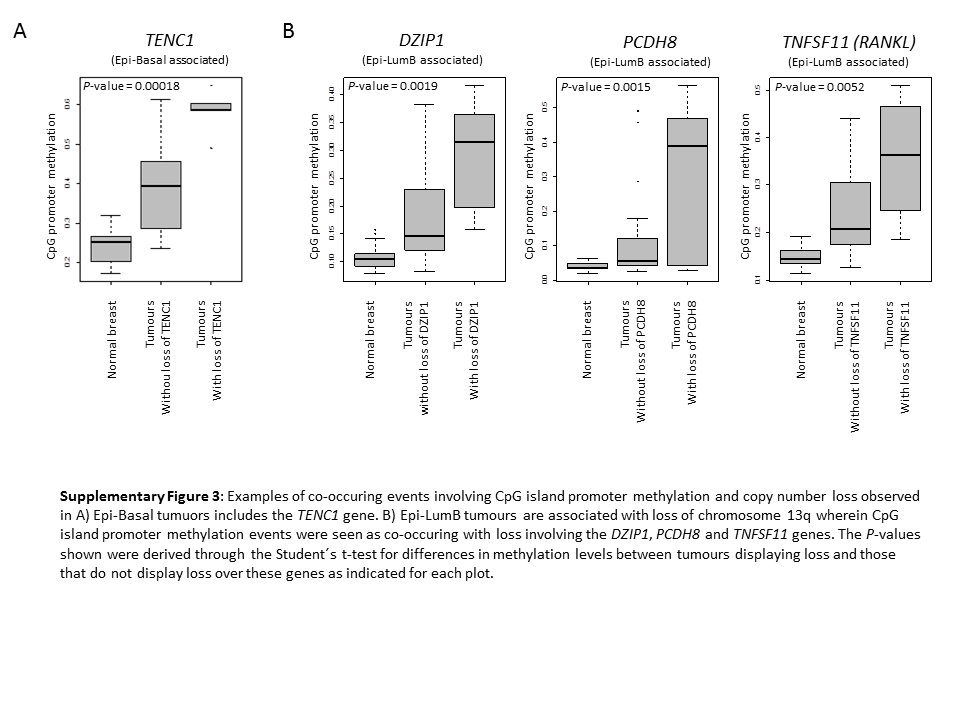


**Supplementary Figure 3:** Examples of co-occurring events involving CpG promoter methylation and copy number loss (hypothetically reflecting loss of one allele and promoter methylation of the retained allele). A) TENC1 promoter methylation is frequently observed in association with copy number loss in Epi-Basal tumors. B) Epi-LumB tumors frequently display loss of chromosome 13q wherein CpG island promoter methylation events can be seen as co-occurring events in DZIP1, PCDH8 and TNFSF11. The P-values displayed are those from Student’s t tests for differences in methylation levels between tumors with and without loss of genes indicated in each plot.


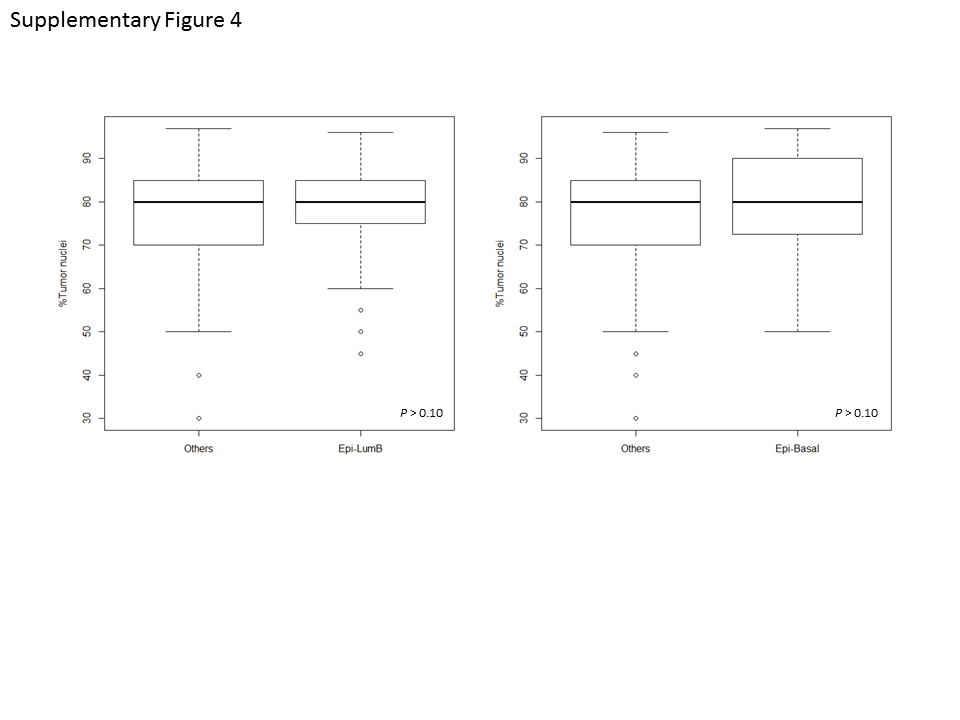


**Supplementary Figure 4:** Analysis of the TCGA cohort where information on the percentage of tumor cell nuclei per sample was available (reflecting tumor cell content of the sample). No differences were detected in comparing the %tumor nucei of Epi-LumB tumor to that of other tumors (left panel) or when comparing %tumor nuclei in Epi-Basal tumors to that of other tumors (right panel). The *P*-values were obtained from the Student’s t-test (two-tailed).

**
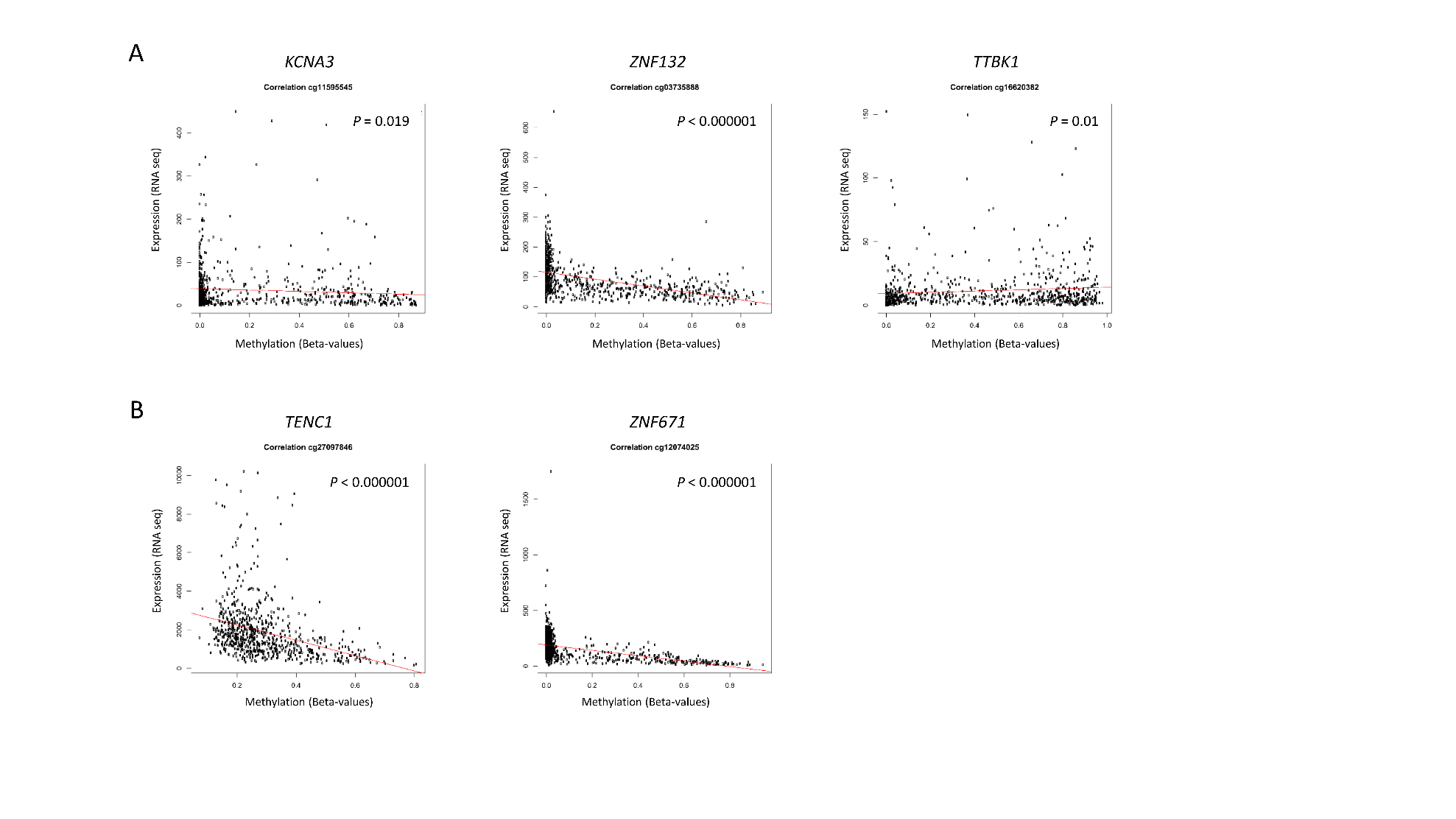
**

**Supplementary Figure 5:** CpG promoter methylation of KCNA3, ZNF132, TTBK1, TENC1 and ZNF671 analyzed in relation with expression. The data are based on Illumina RNA sequencing and Infinium methylation arrays derived from the Cancer Genome Atlas (TCGA). The *P*-values displayed were derived from hypothesis testing of the Pearson´s correlation coefficient using the cor.test function in R.

**Supplementary Table 1**: The relation between overall methylation patterns (as determined through the definition of distinct clusters) analysed in relation to expression-based subtype definitions.

|  | **LumA** | **LumB** | **HER2** | **5NP*** | **Basal-like** | **Unknown** | **Total** |
| --- | --- | --- | --- | --- | --- | --- | --- |
| **Cluster 1** | 1 (14.3%) | 5 (71.4%) | 1 (14.3%) | 0 | 0 | 0 | 7 |
| **Cluster 2** | 1 (6.7%) | 0 | 1 (6.7%) | 0 | 11 (73.3%) | 2 (13.3%) | 15 |
| **Cluster 3** | 4 (100%) | 0 | 0 | 0 | 0 | 0 | 4 |
| *Non-Clustered* | 6 (42.9%) | 2 (14.3%) | 1 (7.1%) | 1 (7.1%) | 3 (21.4%) | 1 (7.1%) | 14 |
|  |  |  |  |  |  |  |  |
|  |  |  |  |  |  |  | *X*^2^-squared=40.6 |
|  |  |  |  |  |  |  | *P*=0.0004 |

*5NP (five negative phenotype; i.e. negativity for ER, PR, HER2, CK5/6 and EGFR)

**Supplementary Table 2**: Subtype-specific CpG’s.

| **LumB-specific CpG methylated markers:** | | | | | |
| --- | --- | --- | --- | --- | --- |
| TargetID | CHR | MAPINFO | UCSC_REFGENE_NAME | UCSC_REFGENE_GROUP | RELATION_TO_UCSC_CPG_ISLAND |
| cg00043819 | 4 | 185942529 |  |  | Island |
| cg00128702 | 18 | 905156 | ADCYAP1;ADCYAP1 | 5'UTR;TSS200 | Island |
| cg00266322 | 17 | 72889549 | FADS6 | 1stExon | Island |
| cg00462168 | 15 | 79724794 | KIAA1024 | TSS200 | Island |
| cg00544449 | 15 | 79724802 | KIAA1024 | TSS200 | Island |
| cg00624404 | 6 | 106442588 |  |  | Island |
| cg00690148 | 2 | 119916017 | C1QL2;C1QL2 | 1stExon;5'UTR | Island |
| cg00690431 | 1 | 186649354 | PTGS2 | Body | Island |
| cg00910695 | 8 | 109095568 | RSPO2;RSPO2 | 1stExon;5'UTR | Island |
| cg01349775 | 6 | 166666838 |  |  | Island |
| cg01447112 | 7 | 6703803 |  |  | Island |
| cg01495122 | 8 | 41167087 | SFRP1 | TSS200 | Island |
| cg01508023 | 4 | 82136116 |  |  | Island |
| cg01532168 | 8 | 140715557 | KCNK9 | TSS1500 | Island |
| cg01565320 | 4 | 42399851 | SHISA3 | TSS200 | Island |
| cg01923218 | 11 | 93063888 | CCDC67;CCDC67 | 1stExon;5'UTR | Island |
| cg01926877 | 15 | 96953051 |  |  | Island |
| cg02062480 | 6 | 108487388 | NR2E1;NR2E1 | 5'UTR;1stExon | Island |
| cg02229993 | 1 | 166134699 | FAM78B | Body | Island |
| cg02262056 | 12 | 66136094 |  |  | Island |
| cg02449821 | 16 | 89641296 | CPNE7;CPNE7 | TSS1500;TSS1500 | Island |
| cg02527669 | 2 | 220417030 | OBSL1 | Body | Island |
| cg02722188 | 4 | 55099058 | PDGFRA | 5'UTR | Island |
| cg02813298 | 19 | 863136 | CFD | Body | Island |
| cg02958321 | 3 | 182972048 | MCF2L2;B3GNT5 | Body;5'UTR | Island |
| cg03133266 | 22 | 23908776 |  |  | Island |
| cg03202804 | 4 | 154710353 | SFRP2 | TSS200 | Island |
| cg03308628 | 12 | 95942287 | USP44;USP44 | 5'UTR;5'UTR | Island |
| cg03378876 | 20 | 36889157 | KIAA1755;KIAA1755 | 5'UTR;1stExon | Island |
| cg04050867 | 8 | 109095572 | RSPO2;RSPO2 | 1stExon;5'UTR | Island |
| cg04125371 | 15 | 48937213 | FBN1 | 5'UTR | Island |
| cg04141813 | 16 | 216450 | HBM | Body | Island |
| cg04156369 | 1 | 101702519 | S1PR1;S1PR1 | 1stExon;5'UTR | Island |
| cg04275490 | 4 | 54967423 | GSX2 | Body | Island |
| cg04353095 | 3 | 140770588 | SPSB4 | TSS200 | Island |
| cg04588165 | 17 | 35300874 |  |  | Island |
| cg04591034 | 5 | 178017937 | COL23A1 | TSS1500 | S_Shore |
| cg04638468 | 4 | 122686453 | TMEM155;LOC100192379 | TSS200;Body | Island |
| cg04701034 | 14 | 52536175 | NID2 | TSS1500 | Island |
| cg04770504 | 7 | 24797363 | DFNA5;DFNA5;DFNA5;DFNA5;DFNA5 | 1stExon;TSS1500;1stExon;5'UTR;5'UTR | Island |
| cg04838747 | 1 | 92950450 | GFI1;GFI1;GFI1 | 5'UTR;5'UTR;TSS1500 | Island |
| cg04912999 | 3 | 142682652 | PAQR9 | TSS1500 | Island |
| cg04974290 | 17 | 5001047 |  |  | Island |
| cg04976324 | 1 | 214162545 | PROX1 | 5'UTR | Island |
| cg05196820 | 4 | 54967018 | GSX2 | 1stExon | Island |
| cg05299793 | 14 | 63512588 | KCNH5;KCNH5;KCNH5 | 5'UTR;TSS1500;TSS1500 | Island |
| cg05375728 | 1 | 58715539 | DAB1 | 5'UTR | Island |
| cg05377226 | 1 | 171810910 | DNM3;DNM3 | 1stExon;1stExon | Island |
| cg05386493 | 3 | 138665654 | FOXL2;C3orf72;FOXL2 | 1stExon;TSS1500;5'UTR | Island |
| cg05478631 | 6 | 166581538 | T | 5'UTR | Island |
| cg05542262 | 5 | 80256535 | RASGRF2 | TSS200 | Island |
| cg05728754 | 12 | 47225326 |  |  | Island |
| cg05743885 | 7 | 27196365 | HOXA7 | TSS200 | Island |
| cg05949903 | 16 | 21831636 | RRN3P1 | TSS1500 | Island |
| cg06088324 | 16 | 19895321 | GPRC5B | 5'UTR | Island |
| cg06094642 | 17 | 35290307 |  |  | N_Shore |
| cg06159352 | 7 | 37487613 | ELMO1 | 5'UTR | Island |
| cg06263193 | 18 | 28622912 | DSC3;DSC3 | TSS200;TSS200 | Island |
| cg06312813 | 4 | 74964493 | CXCL2 | Body | N_Shore |
| cg06319822 | 16 | 215960 | HBM | TSS200 | Island |
| cg06375628 | 4 | 41883879 |  |  | Island |
| cg06377278 | 1 | 25256369 | RUNX3;RUNX3;RUNX3 | 1stExon;Body;5'UTR | Island |
| cg06576393 | 10 | 99531416 | SFRP5 | 1stExon | Island |
| cg06704122 | 2 | 119604702 | EN1 | 1stExon | N_Shore |
| cg06785999 | 14 | 60975964 | SIX6;SIX6 | 1stExon;5'UTR | Island |
| cg07012770 | 18 | 2847301 | EMILIN2 | 1stExon | Island |
| cg07212778 | 2 | 119916510 | C1QL2 | TSS200 | Island |
| cg07213036 | 13 | 28542727 | CDX2 | 1stExon | Island |
| cg07320646 | 7 | 24796981 | DFNA5;DFNA5;DFNA5 | 5'UTR;5'UTR;5'UTR | Island |
| cg07448795 | 22 | 21368419 | P2RX6;P2RX6;MGC16703 | TSS1500;TSS1500;Body | Island |
| cg07482935 | 7 | 103630013 | RELN;RELN | TSS200;TSS200 | Island |
| cg07485357 | 14 | 24803679 | ADCY4 | Body | Island |
| cg07491495 | 15 | 93631578 | RGMA;RGMA;RGMA | Body;5'UTR;1stExon | Island |
| cg07697895 | 7 | 116963259 | WNT2;WNT2;WNT2 | Body;5'UTR;1stExon | Island |
| cg07763047 | 5 | 160974837 | GABRB2;GABRB2 | 5'UTR;5'UTR | Island |
| cg07846220 | 18 | 7117680 | LAMA1 | 1stExon | Island |
| cg08217024 | 14 | 48145108 | MDGA2 | TSS1500 | Island |
| cg08393070 | 16 | 215791 | HBM | TSS200 | Island |
| cg08663159 | 4 | 101111872 | DDIT4L | TSS1500 | S_Shore |
| cg08774368 | 3 | 142839578 | CHST2 | 5'UTR | Island |
| cg08858437 | 3 | 142838938 | CHST2;CHST2 | 5'UTR;1stExon | Island |
| cg09010671 | 13 | 79177763 | POU4F1 | TSS200 | Island |
| cg09092054 | 22 | 19511987 | CLDN5;CLDN5 | 1stExon;Body | Island |
| cg09276565 | 6 | 99281178 | POU3F2 | TSS1500 | Island |
| cg09774787 | 16 | 55690381 | SLC6A2 | TSS200 | Island |
| cg10074544 | 4 | 110223700 | COL25A1;COL25A1;COL25A1;COL25A1 | 1stExon;5'UTR;5'UTR;1stExon | Island |
| cg10086212 | 12 | 49484169 | DHH | Body | Island |
| cg10162522 | 14 | 63512760 | KCNH5;KCNH5;KCNH5 | 5'UTR;TSS1500;TSS1500 | Island |
| cg10406295 | 8 | 41167113 | SFRP1 | TSS200 | Island |
| cg10454246 | 4 | 41883890 |  |  | Island |
| cg10541864 | 4 | 174450722 | HAND2;NBLA00301;HAND2 | 5'UTR;TSS1500;1stExon | Island |
| cg10641721 | 3 | 138665006 | FOXL2;C3orf72 | 1stExon;TSS1500 | Island |
| cg10679277 | 11 | 128392708 | ETS1;ETS1;ETS1 | Body;TSS1500;TSS1500 | S_Shore |
| cg10725720 | 6 | 55039232 | HCRTR2;HCRTR2 | 5'UTR;1stExon | Island |
| cg10730712 | 5 | 178017827 | COL23A1 | TSS1500 | S_Shore |
| cg10732215 | 10 | 22625465 |  |  | Island |
| cg10790429 | 12 | 106974623 |  |  | Island |
| cg10827893 | 1 | 214161368 | PROX1 | TSS1500 | Island |
| cg11018723 | 1 | 25256939 | RUNX3;RUNX3 | Body;TSS200 | Island |
| cg11158057 | 1 | 239549798 |  |  | N_Shore |
| cg11256387 | 5 | 178017879 | COL23A1 | TSS1500 | S_Shore |
| cg11722699 | 18 | 28622813 | DSC3;DSC3 | TSS200;TSS200 | Island |
| cg11831981 | 15 | 51973661 | SCG3;SCG3;SCG3;SCG3 | 5'UTR;1stExon;5'UTR;1stExon | Island |
| cg12002303 | 15 | 68113478 |  |  | Island |
| cg12615766 | 8 | 59058273 | FAM110B | 5'UTR | Island |
| cg12628659 | 15 | 79576216 | ANKRD34C | 5'UTR | Island |
| cg12631351 | 5 | 80256738 | RASGRF2 | 1stExon | Island |
| cg12655190 | 17 | 35290523 |  |  | Island |
| cg12691866 | 6 | 43211491 | TTBK1 | 5'UTR | S_Shore |
| cg12768681 | 1 | 20879547 | FAM43B | 1stExon | Island |
| cg12845520 | 8 | 99076734 | C8orf47;C8orf47 | TSS200;TSS200 | N_Shore |
| cg12874092 | 10 | 17271519 | VIM | Body | Island |
| cg13104713 | 1 | 46632811 |  |  | Island |
| cg13259296 | 2 | 24232884 | MFSD2B | TSS200 | Island |
| cg13434638 | 15 | 68723690 | ITGA11 | Body | Island |
| cg13592399 | 14 | 52535758 | NID2;NID2 | 5'UTR;1stExon | Island |
| cg13631572 | 14 | 24803903 | ADCY4 | TSS200 | Island |
| cg13768269 | 9 | 114140 |  |  | Island |
| cg13801416 | 7 | 134143919 | AKR1B1 | TSS200 | Island |
| cg13829550 | 14 | 74893112 | TMEM90A | TSS1500 | Island |
| cg13849378 | 1 | 241520331 | RGS7;RGS7 | 5'UTR;1stExon | Island |
| cg13849454 | 9 | 14348844 |  |  | Island |
| cg13879483 | 12 | 95942907 | USP44;USP44 | TSS1500;5'UTR | Island |
| cg13930300 | 1 | 92950265 | GFI1;GFI1;GFI1 | 5'UTR;5'UTR;TSS1500 | Island |
| cg13969001 | 1 | 18958084 | PAX7;PAX7;PAX7;PAX7;PAX7;PAX7 | 5'UTR;1stExon;5'UTR;1stExon;1stExon;5'UTR | Island |
| cg13986130 | 1 | 186649330 | PTGS2 | Body | Island |
| cg14058647 | 9 | 88137909 |  |  | Island |
| cg14169333 | 9 | 96721468 |  |  | Island |
| cg14192957 | 9 | 133536793 |  |  | Island |
| cg14236735 | 9 | 133536345 |  |  | Island |
| cg14400498 | 14 | 85999933 | FLRT2 | 5'UTR | Island |
| cg14557534 | 3 | 27763581 | EOMES | 1stExon | Island |
| cg14704758 | 5 | 146888890 |  |  | Island |
| cg14777772 | 4 | 55098130 | PDGFRA | 5'UTR | Island |
| cg14866200 | 4 | 42399843 | SHISA3 | TSS200 | Island |
| cg15110403 | 19 | 17392923 | ANKLE1 | Body | Island |
| cg15344419 | 10 | 131769581 |  |  | Island |
| cg15562912 | 3 | 140770617 | SPSB4 | TSS200 | Island |
| cg15584445 | 12 | 130527046 | LOC100190940 | TSS200 | Island |
| cg15684724 | 8 | 67875033 |  |  | Island |
| cg15839448 | 8 | 41166530 | SFRP1 | 1stExon | Island |
| cg15871441 | 2 | 24232886 | MFSD2B | TSS200 | Island |
| cg15890274 | 11 | 65601265 | SNX32 | TSS200 | Island |
| cg15994026 | 4 | 15780306 | CD38 | Body | Island |
| cg16195091 | 17 | 76227996 | LOC283999 | Body | N_Shore |
| cg16325777 | 10 | 43250569 |  |  | Island |
| cg16573266 | 6 | 106442911 |  |  | Island |
| cg16781647 | 2 | 73151595 | EMX1 | Body | Island |
| cg16898239 | 17 | 74070682 | GALR2 | TSS1500 | Island |
| cg17448335 | 5 | 76249776 | CRHBP | Body | Island |
| cg17526573 | 2 | 50574708 | NRXN1;NRXN1;NRXN1;NRXN1 | Body;1stExon;5'UTR;Body | Island |
| cg17609931 | 4 | 174443529 |  |  | Island |
| cg17619823 | 8 | 37823475 | ADRB3 | 1stExon | Island |
| cg17627617 | 3 | 142682682 | PAQR9 | TSS1500 | Island |
| cg17816908 | 8 | 41167109 | SFRP1 | TSS200 | Island |
| cg17831269 | 1 | 6209018 | CHD5 | Body | Island |
| cg18061259 | 12 | 106977641 | RFX4 | Body | Island |
| cg18172881 | 3 | 136538801 | TMEM22;TMEM22;TMEM22 | 5'UTR;5'UTR;5'UTR | Island |
| cg18209212 | 12 | 130646256 | FZD10 | TSS1500 | Island |
| cg18392016 | 6 | 117591684 | VGLL2;VGLL2 | Body;Body | Island |
| cg18468394 | 5 | 170736021 | TLX3 | TSS1500 | Island |
| cg18475643 | 1 | 46956496 |  |  | Island |
| cg18529845 | 2 | 31806042 | SRD5A2 | TSS200 | Island |
| cg18639233 | 14 | 60975846 | SIX6 | TSS200 | Island |
| cg18683604 | 14 | 51561293 | TRIM9;TRIM9 | 1stExon;1stExon | Island |
| cg18740893 | 3 | 136538934 | TMEM22;TMEM22;TMEM22 | 5'UTR;5'UTR;5'UTR | Island |
| cg18750167 | 6 | 30449172 |  |  |  |
| cg18800085 | 1 | 167599719 | RCSD1 | Body | Island |
| cg18917544 | 6 | 99281070 |  |  | Island |
| cg19003797 | 6 | 137819267 |  |  | Island |
| cg19127283 | 3 | 32859407 | TRIM71 | TSS200 | Island |
| cg19178853 | 4 | 174450408 | NBLA00301;HAND2 | TSS1500;1stExon | Island |
| cg19380001 | 1 | 50889427 | DMRTA2 | TSS1500 | Island |
| cg19456540 | 14 | 60976285 | SIX6 | 1stExon | Island |
| cg19741945 | 3 | 32859587 | TRIM71 | 1stExon | Island |
| cg19806849 | 1 | 231297555 | TRIM67 | TSS1500 | N_Shore |
| cg20058043 | 12 | 130646370 | FZD10 | TSS1500 | Island |
| cg20387387 | 8 | 144511212 |  |  | Island |
| cg20642710 | 4 | 81951951 | BMP3 | TSS200 | Island |
| cg20803857 | 2 | 107502615 | ST6GAL2;ST6GAL2;ST6GAL2 | TSS200;5'UTR;5'UTR | Island |
| cg20944305 | 3 | 152553673 | P2RY1 | 1stExon | Island |
| cg21039778 | 2 | 20866231 | GDF7 | TSS200 | Island |
| cg21176643 | 12 | 106979874 | RFX4 | Body | Island |
| cg21517947 | 8 | 41167107 | SFRP1 | TSS200 | Island |
| cg21534423 | 19 | 24154607 |  |  | Island |
| cg21858380 | 2 | 119916486 | C1QL2 | TSS200 | Island |
| cg21872764 | 22 | 19510977 | CLDN5;CLDN5;CLDN5 | 1stExon;3'UTR;3'UTR | Island |
| cg21885046 | 12 | 130647004 | FZD10 | TSS200 | Island |
| cg22489498 | 1 | 214162711 | PROX1 | 5'UTR | Island |
| cg22686881 | 5 | 170737255 | TLX3 | Body | Island |
| cg22770135 | 5 | 135701240 | TRPC7;TRPC7;TRPC7 | TSS200;TSS200;TSS200 |  |
| cg22868282 | 12 | 47224954 |  |  | Island |
| cg23015696 | 3 | 189838112 | LEPREL1;LEPREL1 | 5'UTR;1stExon | Island |
| cg23209255 | 3 | 154797917 | MME;MME;MME;MME | TSS200;5'UTR;TSS200;5'UTR | Island |
| cg23220551 | 2 | 24232889 | MFSD2B | TSS200 | Island |
| cg23260547 | 1 | 214161371 | PROX1 | TSS1500 | Island |
| cg23316253 | 10 | 8076277 |  |  | Island |
| cg23528400 | 3 | 32859438 | TRIM71 | TSS200 | Island |
| cg23807354 | 1 | 92950249 | GFI1;GFI1;GFI1 | 5'UTR;5'UTR;TSS1500 | Island |
| cg23815582 | 1 | 220101945 | SLC30A10;SLC30A10 | 5'UTR;1stExon | Island |
| cg23965689 | 17 | 42394004 | RUNDC3A;RUNDC3A;RUNDC3A | Body;Body;Body | S_Shore |
| cg23992410 | 1 | 214160848 | PROX1 | TSS1500 | Island |
| cg24071532 | 17 | 32909042 | TMEM132E | Body | Island |
| cg24083324 | 1 | 214162604 | PROX1 | 5'UTR | Island |
| cg24158594 | 8 | 60030763 | TOX | Body | Island |
| cg24319902 | 8 | 41166990 | SFRP1 | TSS200 | Island |
| cg24434959 | 3 | 27763102 | EOMES | 1stExon | Island |
| cg24631482 | 2 | 42329446 |  |  | Island |
| cg24645214 | 8 | 54789978 | RGS20 | Body | Island |
| cg24657817 | 4 | 42153708 | BEND4;BEND4 | Body;Body | Island |
| cg24718971 | 5 | 80256166 | RASGRF2 | TSS1500 | Island |
| cg24843474 | 1 | 241520598 | RGS7 | TSS200 | Island |
| cg24942226 | 6 | 99281500 | POU3F2 | TSS1500 | Island |
| cg25088758 | 4 | 110223598 | COL25A1;COL25A1;COL25A1;COL25A1 | 1stExon;5'UTR;5'UTR;1stExon | Island |
| cg25248750 | 8 | 99077132 | C8orf47;C8orf47 | Body;Body | Island |
| cg25274503 | 7 | 116140128 | CAV2;CAV2 | Body;Body | Island |
| cg25317664 | 1 | 220101962 | SLC30A10;SLC30A10 | 5'UTR;1stExon | Island |
| cg25789861 | 18 | 28622940 | DSC3;DSC3 | TSS200;TSS200 | Island |
| cg25859468 | 7 | 15726166 | MEOX2;MEOX2 | 1stExon;5'UTR |  |
| cg26084529 | 4 | 155664993 | LRAT | TSS200 | Island |
| cg26261793 | 1 | 171810543 | DNM3;DNM3 | TSS200;TSS200 | Island |
| cg26279550 | 12 | 66582805 | IRAK3;IRAK3 | TSS200;TSS200 | Island |
| cg26415547 | 12 | 66583048 | IRAK3;IRAK3;IRAK3;IRAK3 | 1stExon;1stExon;5'UTR;5'UTR | Island |
| cg26464221 | 5 | 119801431 | PRR16 | 5'UTR | S_Shore |
| cg26861703 | 18 | 28622918 | DSC3;DSC3 | TSS200;TSS200 | Island |
| cg26946821 | 17 | 35292390 |  |  | Island |
| cg26983469 | 10 | 17271051 | VIM | 5'UTR | Island |
| cg26989202 | 8 | 99076837 | C8orf47;C8orf47;C8orf47;C8orf47 | 1stExon;5'UTR;1stExon;5'UTR | Island |
| cg27096779 | 7 | 15726466 | MEOX2 | TSS200 |  |
| cg27363327 | 6 | 43211208 | TTBK1 | TSS200 | Island |
| cg27398263 | 13 | 79177700 | POU4F1 | TSS200 | Island |
| cg27442308 | 13 | 95364675 | SOX21 | TSS1500 | Island |
| cg27498114 | 8 | 37823292 | ADRB3 | 1stExon | Island |
| cg27504292 | 12 | 124779532 | FAM101A | 5'UTR | N_Shore |
| cg27504802 | 1 | 231298651 | TRIM67 | TSS200 | Island |
| cg27579953 | 12 | 72667150 | LOC283392;TRHDE;LOC283392 | Body;1stExon;Body | Island |
| cg27602263 | 18 | 56936434 | RAX | Body | Island |
| cg27636310 | 1 | 46632696 |  |  | Island |
| cg27649239 | 15 | 68120393 | LBXCOR1 | Body | Island |
|  |  |  |  |  |  |
| **LumB-specific CpG hypomethylated markers:** | | | | | |
| TargetID | CHR | MAPINFO | UCSC_REFGENE_NAME | UCSC_REFGENE_GROUP | RELATION_TO_UCSC_CPG_ISLAND |
| cg03539903 | 20 | 53088317 |  |  | N_Shelf |
| cg08847382 | 1 | 209852361 |  |  | S_Shelf |
| cg12871652 | 12 | 78581770 | NAV3 | Body |  |
| cg13451937 | 17 | 67710309 |  |  |  |
| cg13606025 | 17 | 68070495 | KCNJ16;KCNJ16 | TSS1500;TSS1500 |  |
| cg16342597 | 17 | 26419687 | NLK | Body |  |
| cg16717546 | 7 | 24474486 |  |  |  |
| cg18432768 | 17 | 68100565 | KCNJ16;KCNJ16;KCNJ16 | 5'UTR;TSS1500;5'UTR |  |
| cg20110978 | 7 | 24837854 | OSBPL3;OSBPL3;OSBPL3;OSBPL3 | 3'UTR;3'UTR;3'UTR;3'UTR |  |
| cg20436458 | 2 | 68149422 |  |  |  |
| cg21871080 | 17 | 68100975 | KCNJ16;KCNJ16;KCNJ16 | 5'UTR;TSS200;5'UTR |  |
| cg23653492 | 12 | 130590137 |  |  |  |
| cg24310711 | 17 | 70026605 |  |  |  |
| cg25325053 | 20 | 58533022 | CDH26 | TSS1500 |  |
| cg26712096 | 7 | 24798855 | DFNA5;DFNA5 | TSS1500;TSS1500 | S_Shore |
|  |  |  |  |  |  |
| **Basal-like specific CpG methylated markers:** | | | | | |
| TargetID | CHR | MAPINFO | UCSC_REFGENE_NAME | UCSC_REFGENE_GROUP | RELATION_TO_UCSC_CPG_ISLAND |
| cg00145419 | 13 | 110966036 | COL4A2 | Body | Island |
| cg00428457 | 2 | 119887680 |  |  |  |
| cg01358444 | 6 | 56406084 | DST;DST;DST;DST;DST | Body;Body;Body;Body;Body | N_Shore |
| cg01502588 | 2 | 119615889 |  |  | N_Shore |
| cg02104952 | 1 | 201481480 |  |  |  |
| cg02124912 | 1 | 202091933 | GPR37L1 | TSS200 |  |
| cg02941008 | 1 | 20810527 | CAMK2N1 | Body | Island |
| cg03202693 | 13 | 113637903 | MCF2L;MCF2L | Body;Body | S_Shelf |
| cg03215105 | 19 | 58238600 | ZNF671 | Body | Island |
| cg03325407 | 1 | 114423726 | BCL2L15 | 3'UTR |  |
| cg03633268 | 8 | 30284255 | RBPMS;RBPMS;RBPMS;RBPMS | Body;Body;Body;Body |  |
| cg04192393 | 15 | 63673286 | CA12;CA12 | Body;Body | N_Shore |
| cg04213746 | 10 | 8106003 | GATA3;GATA3 | Body;Body |  |
| cg04238758 | 8 | 74225117 | RDH10 | Body |  |
| cg04246144 | 5 | 140739501 | PCDHGA4;PCDHGA2;PCDHGB2;PCDHGA1;PCDHGB1;PCDHGB2;PCDHGA3 | Body;Body;TSS1500;Body;Body;TSS1500;Body | N_Shore |
| cg04874286 | 12 | 95477853 | FGD6 | Body |  |
| cg04920032 | 12 | 50262986 | FAIM2 | 3'UTR |  |
| cg05369926 | 17 | 79057147 | BAIAP2;BAIAP2;BAIAP2;BAIAP2 | Body;Body;Body;Body |  |
| cg05483021 | 8 | 54569668 |  |  | Island |
| cg05572417 | 13 | 113637815 | MCF2L;MCF2L | Body;Body | S_Shore |
| cg05859267 | 16 | 77469667 | ADAMTS18 | TSS1500 | S_Shore |
| cg06070625 | 3 | 69812798 | MITF;MITF | Body;TSS200 |  |
| cg06159269 | 1 | 8767347 | RERE;RERE | 5'UTR;5'UTR | S_Shelf |
| cg06303936 | 3 | 172711298 | SPATA16 | Body |  |
| cg06311422 | 6 | 56406336 | DST;DST;DST;DST;DST | Body;Body;Body;Body;Body | N_Shore |
| cg06815715 | 15 | 49148520 | SHC4 | Body |  |
| cg07094847 | 7 | 27160586 | HOXA3;HOXA3 | 5'UTR;TSS1500 | N_Shore |
| cg07242860 | 5 | 140739522 | PCDHGA4;PCDHGA2;PCDHGB2;PCDHGA1;PCDHGB2;PCDHGB1;PCDHGA3 | Body;Body;TSS200;Body;TSS200;Body;Body | N_Shore |
| cg07931024 | 20 | 55925586 | RAE1;RAE1 | TSS1500;TSS1500 | N_Shore |
| cg07945618 | 15 | 99926398 | LRRC28 | 3'UTR |  |
| cg08129759 | 1 | 202091944 | GPR37L1 | TSS200 |  |
| cg08291506 | 15 | 89525620 |  |  |  |
| cg08589212 | 13 | 46098921 | COG3 | Body |  |
| cg10020890 | 16 | 75025753 |  |  |  |
| cg10121113 | 8 | 20112959 | LZTS1 | TSS200 |  |
| cg10128479 | 1 | 156465749 | MEF2D | 5'UTR | N_Shelf |
| cg10298741 | 16 | 73019173 | ZFHX3;ZFHX3 | 5'UTR;5'UTR |  |
| cg10988187 | 12 | 71031813 | PTPRB | TSS1500 |  |
| cg11307417 | 3 | 48310616 | ZNF589 | 3'UTR |  |
| cg11938455 | 6 | 15622032 | DTNBP1;DTNBP1;DTNBP1 | Body;Body;Body |  |
| cg11977686 | 19 | 58238987 | ZNF671;ZNF671 | 1stExon;5'UTR | Island |
| cg12653626 | 1 | 229359488 |  |  |  |
| cg12732998 | 3 | 147131860 | ZIC1 | 3'UTR | S_Shore |
| cg12884406 | 1 | 103573989 | COL11A1;COL11A1;COL11A1;COL11A1;COL11A1;COL11A1;COL11A1;COL11A1 | 5'UTR;5'UTR;1stExon;5'UTR;1stExon;1stExon;5'UTR;1stExon |  |
| cg13107973 | 1 | 215178658 | KCNK2 | TSS1500 |  |
| cg13300939 | 1 | 202091868 | GPR37L1 | TSS200 |  |
| cg13628006 | 10 | 34446040 | PARD3 | Body |  |
| cg13726218 | 13 | 72438249 | DACH1;DACH1;DACH1 | Body;Body;Body | N_Shore |
| cg14685146 | 17 | 42088751 | TMEM101 | 3'UTR | N_Shelf |
| cg15996882 | 17 | 17715815 | SREBF1;SREBF1 | 3'UTR;3'UTR | Island |
| cg16051002 | 5 | 140579160 | PCDHB11 | TSS200 | N_Shore |
| cg16474184 | 12 | 48111507 | P11 | Body |  |
| cg17077762 | 15 | 64996104 | OAZ2 | TSS1500 | S_Shore |
| cg17585421 | 5 | 140536897 | PCDHB17 | Body | Island |
| cg17652792 | 5 | 140562007 | PCDHB16;PCDHB16 | 5'UTR;1stExon | N_Shore |
| cg17835606 | 15 | 48839797 | FBN1 | Body |  |
| cg18003970 | 11 | 12410134 | PARVA | Body |  |
| cg18362770 | 14 | 21966454 | TOX4;METTL3 | 3'UTR;Body |  |
| cg19007141 | 4 | 140808552 | MAML3 | Body |  |
| cg19595107 | 5 | 140734543 | PCDHGA2;PCDHGA4;PCDHGA1;PCDHGA4;PCDHGB1;PCDHGA3 | Body;TSS1500;Body;TSS1500;Body;Body | N_Shore |
| cg19836589 | 20 | 55925603 | RAE1;RAE1 | TSS1500;TSS1500 | N_Shore |
| cg20349803 | 2 | 121036440 | RALB | Body |  |
| cg20366549 | 12 | 6486123 | SCNN1A;SCNN1A | Body;TSS1500 |  |
| cg20688917 | 7 | 27160674 | HOXA3;HOXA3 | 5'UTR;TSS1500 | N_Shore |
| cg21776417 | 3 | 147142415 |  |  | S_Shore |
| cg22332589 | 13 | 110966052 | COL4A2 | Body | Island |
| cg22367250 | 4 | 153199823 |  |  |  |
| cg22701534 | 11 | 19935158 | NAV2;NAV2;NAV2 | Body;Body;Body |  |
| cg22865713 | 6 | 25779897 | SLC17A4 | 3'UTR |  |
| cg22925840 | 2 | 225043045 |  |  |  |
| cg23092956 | 2 | 54846562 | SPTBN1;SPTBN1 | Body;Body |  |
| cg23361862 | 10 | 15256422 | FAM171A1 | Body | S_Shore |
| cg23440816 | 1 | 223948910 | CAPN2;CAPN2 | Body;Body |  |
| cg23756265 | 11 | 127891323 |  |  |  |
| cg23903597 | 17 | 61704154 | MAP3K3;MAP3K3 | Body;Body | S_Shelf |
| cg24016939 | 19 | 58239135 | ZNF671 | TSS200 | S_Shore |
| cg24195796 | 15 | 29362669 | APBA2;APBA2 | Body;Body |  |
| cg24865110 | 17 | 57407818 | YPEL2 | TSS1500 | N_Shore |
| cg25087851 | 11 | 60623918 | GPR44 | TSS1500 | S_Shelf |
| cg25487404 | 8 | 37551945 | ZNF703 | TSS1500 | Island |
| cg25602543 | 20 | 44507533 | ZSWIM3 | 3'UTR |  |
| cg26337998 | 5 | 140749783 | PCDHGA4;PCDHGA2;PCDHGB2;PCDHGA1;PCDHGB3;PCDHGB3;PCDHGA5;PCDHGB1;PCDHGA3 | Body;Body;Body;Body;TSS200;TSS200;Body;Body;Body | N_Shore |
| cg26373541 | 4 | 6644336 | MRFAP1 | 3'UTR | S_Shore |
| cg26777836 | 3 | 26665815 | LRRC3B | 5'UTR | N_Shore |
| cg27024272 | 16 | 86834676 |  |  |  |
| cg27139424 | 11 | 58695093 |  |  |  |
| cg27281882 | 20 | 9820740 | PAK7;PAK7 | TSS1500;TSS1500 | S_Shore |
| cg27313021 | 1 | 202091995 | GPR37L1 | TSS200 |  |
| cg27580905 | 2 | 39335085 | SOS1 | Body |  |
|  |  |  |  |  |  |
| **Basal-like specific CpG hypomethylated markers:** | | | | | |
| TargetID | CHR | MAPINFO | UCSC_REFGENE_NAME | UCSC_REFGENE_GROUP | RELATION_TO_UCSC_CPG_ISLAND |
| cg00038736 | 19 | 38877134 | GGN | Body | Island |
| cg00343906 | 1 | 1696692 | NADK | Body |  |
| cg00577950 | 21 | 45176447 | PDXK | 3'UTR | N_Shore |
| cg00643333 | 22 | 45705037 | FAM118A;FAM118A | TSS1500;TSS200 | N_Shore |
| cg00792053 | 4 | 25097886 |  |  | Island |
| cg01095389 | 11 | 75511563 | DGAT2 | 3'UTR |  |
| cg01288184 | 18 | 20811408 | CABLES1;CABLES1;CABLES1 | Body;Body;Body |  |
| cg01521397 | 20 | 60590872 | TAF4 | Body | S_Shelf |
| cg01727651 | 1 | 111772026 | CHI3L2;CHI3L2;CHI3L2 | Body;TSS1500;Body |  |
| cg02026204 | 22 | 30663007 | OSM | TSS200 |  |
| cg02371401 | 5 | 676784 | TPPP | Body | N_Shore |
| cg02394578 | 1 | 197191366 |  |  |  |
| cg02625638 | 8 | 124218648 | FAM83A;FAM83A | Body;Body | N_Shore |
| cg02742918 | X | 153284103 | IRAK1;IRAK1;IRAK1 | Body;Body;Body | N_Shore |
| cg03332897 | 19 | 740442 | PALM;PALM | Body;Body | Island |
| cg03559389 | 10 | 679322 | DIP2C | Body | Island |
| cg04121731 | 16 | 31886923 | ZNF267 | Body | S_Shore |
| cg04386759 | 6 | 170554795 |  |  |  |
| cg05099145 | 16 | 1796333 | MAPK8IP3;MAPK8IP3 | Body;Body | N_Shore |
| cg05138133 | 16 | 84075503 | SLC38A8 | Body |  |
| cg05303559 | 7 | 158512380 |  |  |  |
| cg05753693 | 8 | 10192930 | MSRA;MSRA;MSRA | Body;Body;Body | S_Shore |
| cg06042504 | 8 | 55087323 |  |  |  |
| cg06373940 | 2 | 128052778 | ERCC3 | TSS1500 | S_Shore |
| cg06527318 | 1 | 25349006 |  |  |  |
| cg06775420 | 16 | 1742245 | HN1L | Body |  |
| cg07139527 | 17 | 80969525 | B3GNTL1 | Body | N_Shore |
| cg07838495 | 17 | 8287763 | RPL26 | TSS1500 | S_Shore |
| cg07856071 | 5 | 15928114 | FBXL7 | Body | Island |
| cg07935320 | 8 | 124218630 | FAM83A;FAM83A | Body;Body | N_Shore |
| cg08086385 | 3 | 72430211 | RYBP | Body |  |
| cg08550317 | 6 | 3850732 | FAM50B | Body | Island |
| cg08613144 | 2 | 133428836 | LYPD1;LYPD1;LYPD1 | 1stExon;5'UTR;TSS1500 | Island |
| cg08701134 | 16 | 69969618 | WWP2;WWP2 | Body;Body |  |
| cg08913523 | 8 | 126649807 |  |  |  |
| cg08944347 | 7 | 76087149 |  |  | N_Shelf |
| cg09010067 | 17 | 30821827 | MYO1D | Body | N_Shore |
| cg09156097 | 5 | 138729283 | LOC389333 | 1stExon | Island |
| cg09476006 | 5 | 138032270 |  |  |  |
| cg10092251 | 13 | 101196790 |  |  |  |
| cg10092607 | 4 | 25097821 |  |  | Island |
| cg10684794 | 19 | 40901963 | PRX;PRX | 3'UTR;Body | N_Shelf |
| cg10851763 | 21 | 43545792 | UMODL1;UMODL1 | Body;Body | N_Shelf |
| cg11005831 | 5 | 15928441 | FBXL7 | Body | Island |
| cg11509491 | 10 | 93976249 | CPEB3 | Body |  |
| cg11683663 | 19 | 855536 | ELANE | Body | Island |
| cg11693986 | 7 | 100805303 |  |  | N_Shore |
| cg11833938 | 5 | 555251 |  |  |  |
| cg11888982 | 8 | 116231420 |  |  |  |
| cg11895696 | 7 | 105283982 | ATXN7L1;ATXN7L1 | Body;Body |  |
| cg11902728 | 19 | 35786580 | MAG;MAG | Body;Body | Island |
| cg12245706 | 2 | 71823484 | DYSF;DYSF;DYSF;DYSF | Body;Body;Body;Body |  |
| cg12600109 | 20 | 62321166 | RTEL1;RTEL1 | Body;Body | Island |
| cg12887985 | 5 | 43020716 |  |  | S_Shelf |
| cg13100449 | 1 | 184836205 | FAM129A | Body |  |
| cg13221899 | 13 | 103426862 | C13orf27 | TSS1500 | Island |
| cg13263472 | 14 | 102593156 | HSP90AA1 | Body |  |
| cg13615592 | 15 | 101420708 | ALDH1A3 | Body | Island |
| cg13656752 | 5 | 171538334 | STK10 | Body |  |
| cg14210094 | 2 | 62888566 |  |  |  |
| cg14533992 | 4 | 25097558 |  |  | N_Shore |
| cg14864276 | 5 | 177713841 | COL23A1 | Body |  |
| cg14950747 | 19 | 3821095 | ZFR2 | Body | Island |
| cg15477144 | 22 | 45705034 | FAM118A;FAM118A | TSS1500;TSS200 | N_Shore |
| cg15545942 | 2 | 9526703 | ASAP2;ASAP2 | Body;Body |  |
| cg15773230 | 4 | 25097661 |  |  | Island |
| cg15809077 | 2 | 112462079 |  |  |  |
| cg16565901 | 17 | 78735596 | RPTOR;RPTOR | Body;Body |  |
| cg16577083 | 16 | 1271153 | CACNA1H;CACNA1H | 3'UTR;3'UTR | S_Shelf |
| cg17029019 | 17 | 1959124 | HIC1;HIC1 | TSS1500;5'UTR | Island |
| cg17389150 | 19 | 2997638 | TLE2;TLE2;TLE2 | 3'UTR;3'UTR;3'UTR |  |
| cg17473898 | 19 | 836679 |  |  | Island |
| cg17939889 | 2 | 240174650 | HDAC4 | Body |  |
| cg18346398 | 6 | 1615992 |  |  | S_Shore |
| cg18428193 | 2 | 27665017 | KRTCAP3;NRBP1;KRTCAP3 | TSS1500;3'UTR;TSS1500 | Island |
| cg18990407 | 3 | 184297380 | EPHB3 | Body | N_Shelf |
| cg19234983 | 1 | 211688020 |  |  | N_Shore |
| cg19254793 | 6 | 44695348 |  |  | Island |
| cg19324997 | 2 | 240169161 | HDAC4 | Body |  |
| cg19465124 | 4 | 184344219 |  |  |  |
| cg19573208 | 20 | 60773564 | GTPBP5 | Body | N_Shelf |
| cg20040765 | 8 | 67358546 | ADHFE1 | Body |  |
| cg20199549 | 10 | 820835 |  |  |  |
| cg20664445 | 17 | 80969547 | B3GNTL1 | Body | N_Shore |
| cg20712980 | 7 | 129305192 | NRF1;NRF1 | Body;Body |  |
| cg20762346 | 6 | 168619344 |  |  |  |
| cg21010202 | 6 | 1615843 |  |  | Island |
| cg21282054 | 17 | 76127608 | TMC8;TMC6 | 5'UTR;5'UTR | Island |
| cg21348406 | 17 | 76361166 |  |  |  |
| cg21938845 | 7 | 75915065 | SRRM3 | Body | S_Shelf |
| cg22194807 | 18 | 32957683 | ZNF396 | TSS1500 | S_Shore |
| cg22308726 | 19 | 4566575 |  |  | Island |
| cg22439792 | 1 | 156613060 | BCAN;BCAN | 5'UTR;5'UTR | S_Shore |
| cg22525069 | 3 | 193587264 |  |  | N_Shore |
| cg22544679 | 6 | 3285299 | SLC22A23;SLC22A23 | Body;Body |  |
| cg22740006 | 11 | 66626141 | PC;PC;LRFN4;PC | Body;Body;1stExon;Body | Island |
| cg22745369 | 6 | 3517953 |  |  |  |
| cg23421509 | 2 | 8978201 | KIDINS220 | TSS1500 | S_Shore |
| cg23590389 | 7 | 157102769 |  |  |  |
| cg23698536 | 8 | 124218573 | FAM83A;FAM83A | Body;Body | N_Shore |
| cg23884187 | 20 | 37275069 | C20orf95 | Body | Island |
| cg24452821 | 11 | 113953812 | ZBTB16;ZBTB16 | Body;Body | Island |
| cg24687806 | 6 | 168132832 |  |  | N_Shelf |
| cg25432975 | 17 | 1959066 | HIC1;HIC1 | TSS1500;5'UTR | Island |
| cg25666403 | 22 | 30662994 | OSM | TSS200 |  |
| cg25887076 | 10 | 5576256 |  |  |  |
| cg26056277 | 2 | 166982925 |  |  |  |
| cg26495711 | 2 | 240169280 | HDAC4 | Body |  |
| cg26664254 | 10 | 131589189 |  |  | N_Shelf |
| cg27109748 | 7 | 2150016 | MAD1L1;MAD1L1;MAD1L1 | Body;Body;Body |  |
| cg27132471 | 15 | 92613280 | SLCO3A1;SLCO3A1 | Body;Body |  |
| cg27344587 | 2 | 63275602 | LOC100132215 | TSS1500 | Island |
| cg27615363 | 5 | 174120078 |  |  |  |

**Supplementary Table 3**: CpG promoter methylation events identified as candidate proxy markers for the novel Epi-Subtypes (i.e. Epi-LumB and Epi-Basal).

| **Epi-LumB associated CpG island promoter methylation events (listing only the top 30 significantly associated genes; only CpG island associated)** | | | | | | | |
| --- | --- | --- | --- | --- | --- | --- | --- |
| **Gene Symbol** | **TargetID´s** | | | | | | |
| BICC1 | cg07857251 | cg19940537 |  |  |  |  |  |
| CHST2 | cg08858437 | cg08774368 |  |  |  |  |  |
| CUGBP2 | cg03813164 | cg12356890 | cg11472279 |  |  |  |  |
| DNM3 | cg26261793 | cg06211893 | cg05377226 |  |  |  |  |
| EOMES | cg24434959 | cg14557534 |  |  |  |  |  |
| FMN2 | cg01535698 | cg25208017 |  |  |  |  |  |
| FSD1 | cg13355047 | cg19752891 | cg07678904 |  |  |  |  |
| GFI1 | cg11785652 | cg11412935 | cg23807354 | cg13930300 | cg20813081 |  |  |
| GLB1L3 | cg21692846 | cg03323292 | cg05621343 |  |  |  |  |
| IGFBP7 | cg11597475 | cg21921384 | cg01959562 |  |  |  |  |
| ITGA4 | cg06952671 | cg21995919 | cg25024074 |  |  |  |  |
| KCNA3 | cg20302133 | cg26013553 | cg11595545 | cg01423964 |  |  |  |
| LHX1 | cg05527869 | cg10043865 | cg10356613 |  |  |  |  |
| LOC100287216 | cg12518410 | cg09392940 |  |  |  |  |  |
| LOC285548 | cg16292168 | cg10461004 |  |  |  |  |  |
| MFSD2B | cg02687055 | cg23220551 |  |  |  |  |  |
| POU4F1 | cg27398263 | cg09010671 |  |  |  |  |  |
| QRFPR | cg19971716 | cg13643914 |  |  |  |  |  |
| RUNX3 | cg06377278 | cg11018723 |  |  |  |  |  |
| SHISA3 | cg06269673 | cg16862295 | cg14866200 | cg01565320 |  |  |  |
| SLC30A10 | cg17721710 | cg25317664 |  |  |  |  |  |
| SNCA | cg08708229 | cg02192967 | cg00119181 | cg20776829 |  |  |  |
| STK33 | cg08788717 | cg00450824 | cg00393798 |  |  |  |  |
| TBX21 | cg26281453 | cg09775582 | cg20568196 |  |  |  |  |
| TM6SF1 | cg26460092 | cg03063639 |  |  |  |  |  |
| TMEM155 | cg10863741 | cg04638468 |  |  |  |  |  |
| TMEM22 | cg22304507 | cg18740893 |  |  |  |  |  |
| TTBK1 | cg27363327 | cg16620382 |  |  |  |  |  |
| VWC2 | cg01893212 | cg02467990 |  |  |  |  |  |
| ZNF132 | cg12042659 | cg03735888 |  |  |  |  |  |
| **Epi-Basal associated CpG promoter methylation events (listing all the 8 significantly associated gene promoter regions; CpG island or non-island associated)** | | | | | | | |
| **Gene Symbol** | **TargetID´s** | | | | | | |
| FAM198B | cg03304437 | cg24811290 | cg12543949 | cg03450635 |  |  |  |
| MARCH11 | cg25092681 | cg00339556 | cg01791874 | cg17030173 | cg17712694 | cg16150752 | cg21901718 |
| PCDHB7 | cg24168884 | cg25412979 |  |  |  |  |  |
| PCDHGA1 | cg22184507 | cg11011625 | cg07242860 |  |  |  |  |
| TENC1 | cg17094065 | cg18037834 |  |  |  |  |  |
| ZNF257 | cg24175803 | cg02912127 |  |  |  |  |  |
| ZNF486 | cg03570035 | cg07227744 | cg24749688 |  |  |  |  |
| ZNF671 | cg12074025 | cg19246110 | cg11977686 | cg08048222 | cg24016939 |  |  |
